# miR–9-5p regulates immunometabolic and epigenetic pathways in **β**-glucan–trained immunity via IDH3**α**

**DOI:** 10.1172/jci.insight.144260

**Published:** 2021-05-10

**Authors:** Haibo Su, Zhongping Liang, ShuFeng Weng, Chaonan Sun, Jiaxin Huang, TianRan Zhang, Xialian Wang, Shanshan Wu, Zhi Zhang, Yiqi Zhang, Qing Gong, Ying Xu

**Affiliations:** 1 The Sixth Affiliated Hospital, GMU-GIBH Joint School of Life Science, Guangzhou Medical University, Guangzhou, China.; 2 State Key Laboratory of Genetic Engineering, Institute of Genetics, School of Life Science, Fudan University, Shanghai, China.; 3 Department of General Surgery, The Sixth Affiliated Hospital, Guangzhou Medical University, Qingyuan, China.

**Keywords:** Immunology, Inflammation, Innate immunity, Monocytes

## Abstract

Trained immunity, induced by β-glucan in monocytes, is mediated by activating metabolic pathways that result in epigenetic rewiring of cellular functional programs; however, molecular mechanisms underlying these changes remain unclear. Here, we report a key immunometabolic and epigenetic pathway mediated by the *miR–9-5p*-isocitrate dehydrogenase 3α (IDH3α) axis in trained immunity. We found that β-glucan–trained *miR–9-5p^–/–^* monocytes showed decreased IL-1β, IL-6, and TNF-α production after LPS stimulation. Trained *miR–9-5p^–/–^* mice produced decreased levels of proinflammatory cytokines upon rechallenge in vivo and had worse protection against *Candida albicans* infection. miR–9-5p targeted IDH3α and reduced α-ketoglutarate (α-KG) levels to stabilize HIF-1α, which promoted glycolysis. Accumulating succinate and fumarate via miR–9-5p action integrated immunometabolic circuits to induce histone modifications by inhibiting KDM5 demethylases. β-Glucan–trained monocytes exhibited low IDH3α levels, and IDH3α overexpression blocked the induction of trained immunity by monocytes. Monocytes with IDH3α variants from autosomal recessive retinitis pigmentosa patients showed a trained immunity phenotype at immunometabolic and epigenetic levels. These findings suggest that miR–9-5p and IDH3α act as critical metabolic and epigenetic switches in trained immunity.

## Graphical abstract

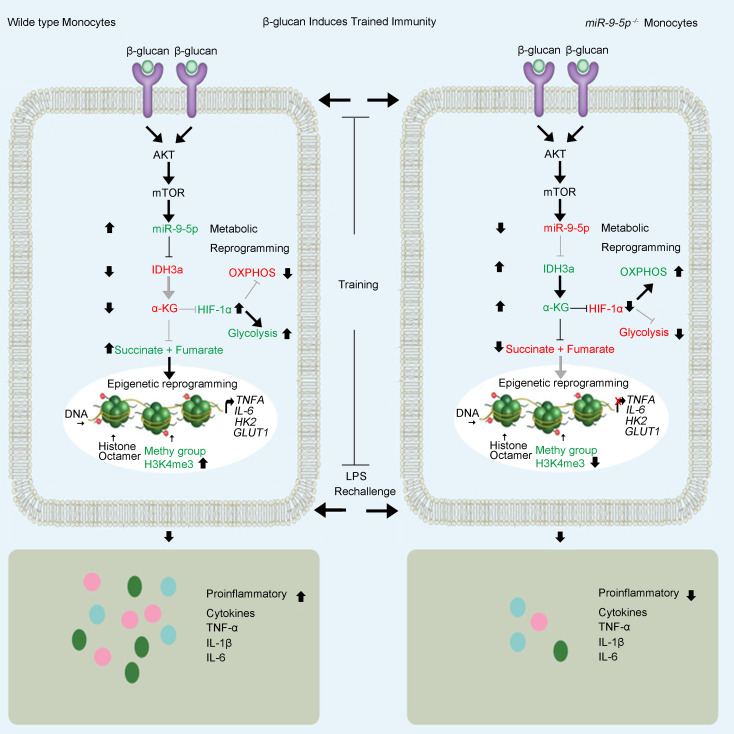

## Introduction

Trained immunity, the nonspecific memory of the innate immune system, has been present by a wide variety of recent — but also older — studies in plants, invertebrates, and mammals (1, 2). Innate immune cells were previously challenged in vitro with microbial components such as β-glucan of the *Candida albicans* (*C*. *albicans*) cell wall or the *Bacillus Calmette-Guérin* (BCG) vaccine and restimulated with the same or different microbial insult a week after training, resulting in increased capacity of the challenged cells to produce cytokines compared with nontrained cells (3, 4). Additionally, when mice were trained in vivo with β-glucan, BCG, or a low dose of *C*. *albicans*, they showed lower mortality after lethal *C*. *albicans* reinfections (5). Trained monocytes have high glucose consumption, lactate production, and NAD^+^/NADH ratio, displaying a shift from oxidative phosphorylation to aerobic glycolysis (Warburg effect), depending on the activation of mTOR through the Dectin-1/Akt/HIF-1α pathway (6). Of note, other metabolites from the tricarboxylic acid (TCA) cycle also play key roles in monocyte remodeling. Fumarate promotes the stabilization of HIF-1α, which are responsible for mounting trained immunity; in contrast, α-KG leads to the hydroxylation of HIF-1α for degradation and impaired monocyte induction of trained immunity (7). Causal to the enhanced inflammatory response in trained immunity is the increased deposition of histone marks that are positively correlated with transcription at the promoters of key immune genes. The promoters of trained immune genes are enriched for H3K4me3 (8). Accumulation of fumarate contributes to trained immunity phenotype by inhibiting KDM5 histone demethylases (responsible for H3K4me3 demethylation) and, thus, influencing epigenetic reprogramming, whereas α-ketoglutarate (α-KG) counteracts this effect (7). However, molecular mechanisms underlying cellular metabolism reprogramming, with a shift from oxidative phosphorylation to aerobic glycolysis and the accumulation of metabolites from the TCA cycle, have not been fully deciphered, thereby impairing understanding of trained immunity.

Isocitrate dehydrogenases (IDHs) are a group of enzymes that catalyze the oxidative decarboxylation of isocitrate (ICT) to α-KG. Three IDH paralogs, localized in different subcellular compartments, show different enzymatic characteristics. IDH1 and IDH2 are homodimeric nicotinamide adenine dinucleotide phosphate–dependent (NADP-dependent) enzymes that reversibly convert ICT to α-KG (9). Previously, IDH1 and IDH2 inhibition was shown to reduce α-KG and NADPH levels and inhibit the α-KG–dependent function of Jumonji-C domain–containing histone lysine residue demethylases, resulting in a global hypermethylation phenotype (10). IDH3 is a nicotinamide adenine dinucleotide–dependent (NAD-dependent) heterotetrameric enzyme that irreversibly catalyzes the conversion of ICT to α-KG in the TCA cycle. Diminished IDH3 activity reduced the ratio of α-KG to succinate and fumarate, which in return, promotes hypoxia-inducible factor 1α–dependent (HIF-1α–dependent) upregulation of glycolytic enzymes and dampens oxidative phosphorylation (10). Addressing whether and how IDH activity is regulated in monocytes trained by β-glucan and whether this regulation involves metabolic and epigenetic rewiring of trained immunity is fundamental.

miRNAs are short, single-stranded RNAs that regulate posttranscriptional mRNA expression by binding to complementary mRNA sequences, resulting in translational repression and gene silencing (11). These noncoding RNAs play critical roles in various physiological processes, such as cell differentiation, cellular rewiring, metabolism reprogramming, and epigenetic modification (12). However, our understanding of the role of miRNAs in modulating trained immunity is limited (13). Here, our aim was to unveil the early transcriptional and metabolic events following monocyte exposed to β-glucan and how the resulting epigenetic changes determine the function of trained monocytes. We showed that miR–9-5p was abundantly expressed in β-glucan–trained monocytes via the Dectin-1/Akt/mTOR/GSK3β pathway and that it contributed to trained immunity via IDH3α.

## Results

### miR–9-5p induction is dependent on the Dectin-1/Akt/mTOR/GSK3β pathway in β-glucan–induced trained immunity.

**Figure 1:**
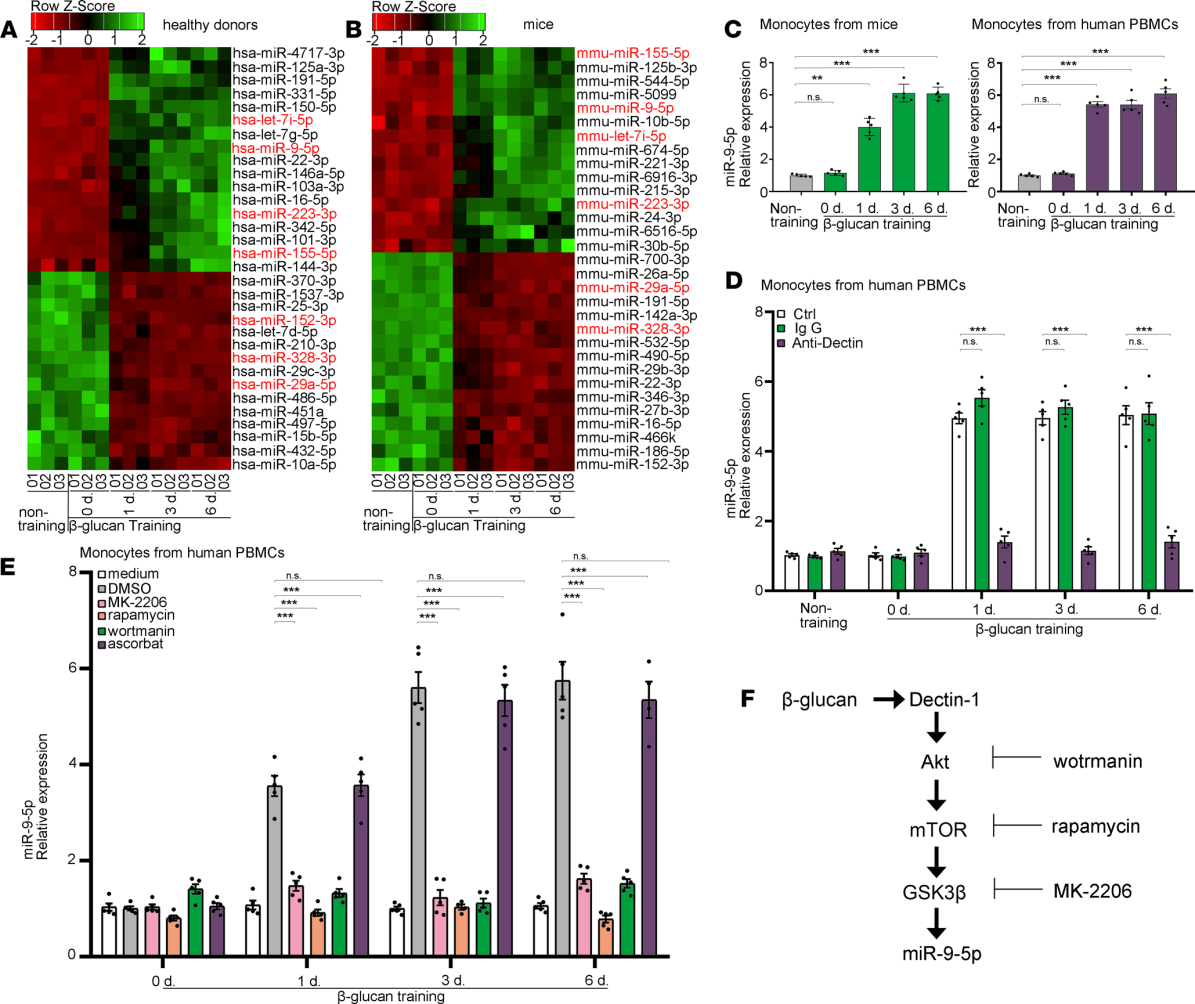
miR–9-5p induction is dependent on the Dectin-1/Akt/mTOR/GSK3β pathway in β-glucan–induced trained immunity. (**A** and **B**) Heatmap shows downregulated (red) and upregulated (green) miRNA from: PBMCs of healthy donors (**A**) and murine PBMCs (**B**) trained with β-glucan. The scale bar at the top ranges from red to green (low to high expression). RNA-seq was performed once in triplicate (*n* = 3). (**C**) miR–9-5p levels were determined using qPCR in β-glucan–trained monocytes at the indicated times (*n* = 3 independent experiments). (**D**) Dectin-1 (20 μg/mL) blocking impaired miR–9-5p expression in β-glucan–trained monocytes (*n* = 3 independent experiments). (**E**) miR–9-5p expression in β-glucan–trained monocytes in the presence of DMSO (control), rapamycin (100 nM), wortmanin (30 mM), MK2206 (10 μm), or ascorbate (50 μM) (*n* = 3 independent experiments). (**F**) Working model illustrating that the increased miR–9-5p induction occurs in a Dectin-1/Akt/mTOR/GSK3β–dependent manner. Human PBMCs from 5 donors; mouse blood mononuclear cells from 10 WT mice. Data represent means ± SEM. **P < 0.01, ***P < 0.001 by 1-way ANOVA/Dunnett’s multiple comparisons test (**C**, **D**, and **E**).

To investigate the effect of miRNA expression on β-glucan–induced trained immunity, we evaluated miRNA expression profiles of β-glucan–trained myeloid cells, including peripheral blood mononuclear cells (PBMCs) of healthy donors, and the blood mononuclear cells of C57BL/6 mice (Supplemental Table 1; supplemental material available online with this article; https://doi.org/10.1172/jci.insight.144260DS1). The heatmap, as a result of miRNA deep sequencing, revealed distinct expression patterns in some miRNAs in β-glucan–trained human and mouse monocytes (Figure 1, A and B). We found that miR–9-5p was upregulated in all β-glucan–trained samples compared with their controls. Since miR–9-5p regulating β-glucan–trained immunity remains uncharacterized, we focused on investigating its role in this event in further study. The upregulation of miR–9-5p was validated using quantitative PCR (qPCR) in monocytes trained with β-glucan (Figure 1C). Induction of miR–9-5p was also found in BCG and IL-1β–trained monocytes but not in IFN-γ–trained cells (Supplemental Figure 1A). Previously, the Dectin-1/Akt/mTOR/ HIF-1α pathway was identified as a central regulatory mechanism for β-glucan–induced trained immunity (6). We found that β-glucan–induced miR–9-5p expression was significantly attenuated in Dectin-1 antibody–blocked monocytes (Figure 1D and Supplemental Figure 1B). To further investigate the signaling involved in induction of miR–9-5p, we trained monocytes with β-glucan in the presence of a selective mTOR inhibitor (rapamycin), Akt inhibitor (wortmannin), or HIF-1α inhibitor (ascorbate). The result showed that Akt and mTOR inhibition significantly reduced miR–9-5p expression in β-glucan–trained monocytes (Figure 1E and Supplemental Figure 1, C and D). In contrast, the miR–9-5p level remained unchanged upon HIF-1α inhibition. Similarly, short hairpin RNA–mediated (shRNA-mediated) silencing of Akt and mTOR expression also robustly decreased miR–9-5p expression in monocytes trained with β-glucan (Supplemental Figure 2, A–D). GSK3β is known to regulate microRNA biogenesis (14). We found that total GSK3β level was increased in monocytes trained with β-glucan dependent on Akt/mTOR function (Supplemental Figure 2, A and C). GSK3β chemical inhibition or loss of function decreased miR–9-5p level in β-glucan–trained monocytes (Figure 1E; Supplemental Figure 1, C and D; and Supplemental Figure 2, E and F). These results suggested that β-glucan–trained immunity induces miR–9-5p expression via the Dectin-1/Akt/mTOR/GSK3β pathway (Figure 1F).

### miR–9-5p promotes β-glucan–induced trained immunity in vitro.

**Figure 2:**
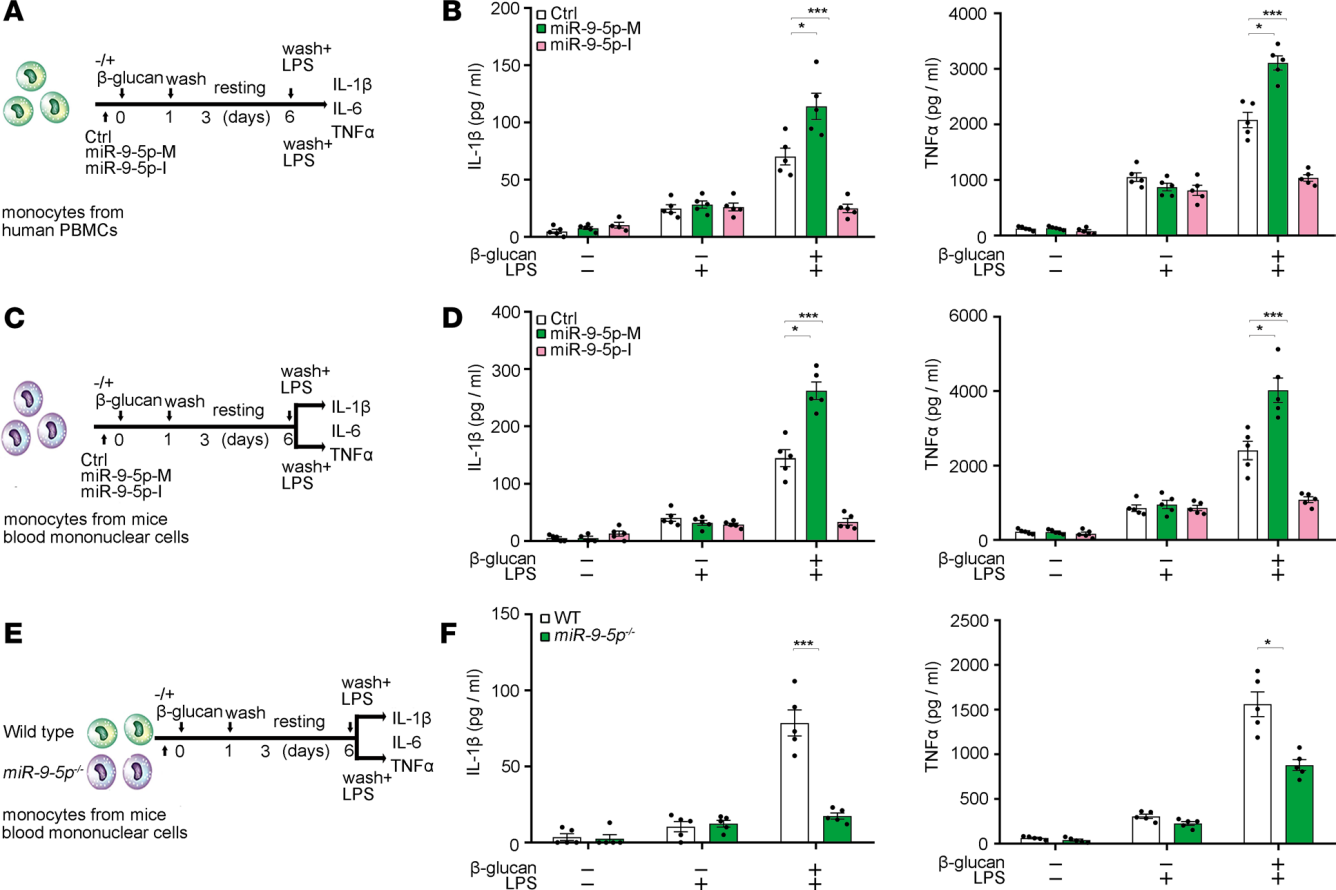
miR–9-5p promotes β-glucan–induced trained immunity in vitro. (**A**, **C**, and **E**) Schematic representation of in vitro trained immunity experimental setup. (**B**, **D**, and **F**) IL-1β and TNF-α production were analyzed in the supernatants of monocytes response to LPS according to **A**, **C**, and **E** (*n* = 3 independent experiments). Human PBMCs from 5 donors; mouse blood mononuclear cells from 10 WT mice or 8 *miR–9-5p^–/–^* mice. Data represent means ± SEM. *P < 0.05, ***P < 0.001 by 1-way ANOVA/Dunnett’s multiple comparisons test (**B** and **D**); *P < 0.05, ***P < 0.001 by 2-tailed Student’s *t* test comparing WT and *miR–9-5p^–/–^* (**F**). miR–9-5p-M, miR–9-5p mimic; miR–9-5p-I, miR–9-5p inhibitor.

To examine the potential involvement of miR–9-5p in β-glucan–triggered trained immunity, we transiently transfected monocytes with a miR–9-5p mimic or inhibitor, trained them with β-glucan for 24 hours, and then evaluated cytokine production in response to LPS (Figure 2, A and C). We found that treatment with the miR–9-5p mimic showed a higher production of IL-1β and TNF-α upon LPS stimulation under β-glucan training compared with the control group, whereas miR–9-5p inhibition significantly reduced the production of these cytokines (Figure 2, B and D). Moreover, β-glucan–trained *miR–9-5p^–/–^* monocytes showed decreased production of these trained immunity–associated cytokines compared with WT cells (Figure 2, E and F). Of note, the miR–9-5p did not affect the cytotoxicity or cell viability under trained conditions (Supplemental Figure 3, A and B). Surface expression of the receptors in trained immunity, including Dectin-1, NOD2, TLR2, and TLR4, were comparable between WT and *miR–9-5p^–/–^* monocytes (Supplemental Figure 3C). These findings indicate that miR–9-5p promotes β-glucan–induced trained immunity in monocytes.

### miR–9-5p deletion impairs trained immunity in vivo.

**Figure 3:**
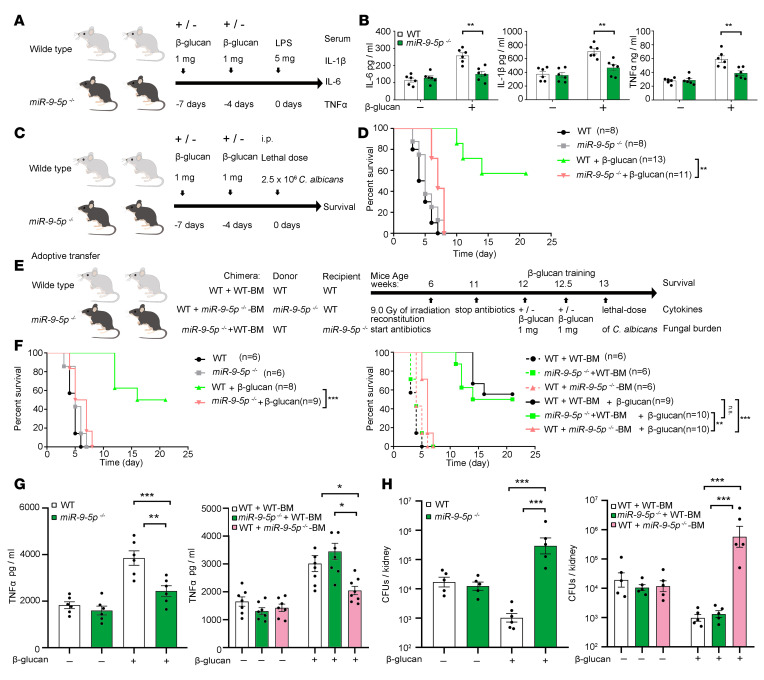
miR–9-5p deletion impairs trained immunity in vivo. (**A**) In vivo training mouse model via 2 i.p. and secondary i.p. β-glucan injections. (**B**) LPS challenge for measuring serum cytokines (*n* = 3 pooled experiments). (**C**) In vivo training mouse model similar to **A** but with secondary *C*. *albicans* lethal infection. (**D**) Survival curve, according to **C**. (**E**) List of radiation chimeras used in this study and experimental design. (**F**) Survival curves of radiation chimeras, WT, and *miR–9-5p^–/–^* mice trained with β-glucan, followed with a lethal *C*. *albican* infection, according to **E**. (**G**) The production of TNF-α was determined in lethally infected monocytes from trained radiation chimeras and WT and *miR–9-5p^–/–^* mice, according to **E**. (**H**) Kidney fungal burden was determined in lethally infected monocytes from trained radiation chimeras and WT and *miR–9-5p^–/–^* mice, according to **E**. In **B**, **G**, and **H**, single dots correspond to individual mice; means ± SEM of 2 or 3 pooled experiments are shown, including 5–6 mice per condition. **P < 0.01 by 2-tailed Student’s *t* test comparing WT and *miR–9-5p^–/–^* (**B**); *P < 0.05, **P < 0.01, ***P < 0.001 by 1-way ANOVA/Tukey’s multiple comparisons test (bottom) (**G** and **H**). A pool of 2 experiments is shown, including 6–13 mice per group as indicated. **P < 0.01, ***P < 0.001 by log-rank test (**D** and **F**).

Induction of trained immunity in vivo provides cross protection against unrelated secondary infections (15). To investigate the role of miR–9-5p in β-glucan trained immunity in vivo, we challenged WT and *miR–9-5p^–/–^* mice with LPS after training with β-glucan, and serum cytokine levels were measured (Figure 3A). Serum levels of IL-1β, IL-6, and TNF-α were significantly decreased in trained *miR–9-5p^–/–^* mice compared with WT mice (Figure 3B), supporting the regulatory role of miR–9-5p upon β-glucan training in vivo. After training with β-glucan, WT and *miR–9-5p^–/–^* mice were i.v. injected with a lethal dose of *C*. *albicans* (Figure 3C). Both WT and *miR–9-5p^–/–^* nontrained mice rapidly succumbed to these infection conditions (Figure 3D), indicating that miR–9-5p is redundant for the primary response to lethal candidiasis. However, the protective response of β-glucan training against lethal *C*. *albicans* infection was significantly attenuated in *miR–9-5p^–/–^* mice compared with WT littermates (Figure 3D). Similarly, the nonlethal dose of *C*. *albicans* training improved the survival of WT mice; in contrast, *miR–9-5p^–/–^* mice were more susceptible to the lethal fungal infection (Supplemental Figure 4, A and B). The production of IL-1β, IL-6, and TNF-α was also inhibited in *miR–9-5p^–/–^* mice (Supplemental Figure 4C), together with an increased renal fungal burden compared with WT (Supplemental Figure 4D). Therefore, miR–9-5p plays a crucial role in inducing protective trained immunity in vivo.

To more specifically examine the effect of miR–9-5p on β-glucan–induced trained immunity in myeloid-derived cells, we used adoptive transfer (16, 17) to generate mice lacking miR–9-5p in myeloid-derived cells. WT and *miR–9-5p^–/–^* mice were lethally irradiated, and their BM reconstituted with either *miR–9-5p^–/–^* or WT BM (Figure 3E). Flow cytometry showed that, 5 weeks after BM transplantation, WBCs were efficiently reconstituted and displayed the donor *miR–9-5p* genotype (Supplemental Figure 4E). Reconstitution was essentially complete for CD11b^+^ cells (>95%; Supplemental Figure 4E). For investigating the reconstitution of resident monocytes, we detected miR–9-5p expression in chimeric monocytes under training condition. The qPCR assay showed that miR–9-5p expression was dramatically increased in monocytes from *miR–9-5p^–/–^* reconstituted with WT BM (*miR–9-5p^–/–^*+WT-BM chimeras; Supplemental Figure 4F), in which monocytes were almost fully derived from the donor BM, whereas parenchymal cells were of the recipient. To determine the compartment in which miR–9-5p deficiency prevents β-glucan–trained immunity, WT and *miR–9-5p^–/–^* mice, as well as the BM transplant groups (radiation chimeras), were i.v. injected with a lethal dose of *C*. *albicans* after β-glucan training (Figure 3E). As a result, *miR–9-5p^–/–^* mice on β-glucan training died after the lethal *C*. *albicans* challenge (Figure 3F). Conversely, *miR–9-5p^–/–^*+WT-BM chimeras were protected from the lethal *C*. *albicans* infection (Figure 3F). TNF-α level was reduced in WT*+miR–9-5p^–/–^*-BM chimeras versus WT+WT-BM and *miR–9-5p^–/–^*+WT-BM (Figure 3G), together with an increased renal fungal burden (Figure 3H). We concluded that resistance to β-glucan–induced immunity in *miR–9-5p^–/–^* mice is mainly due to miR–9-5p absence in resistant monocytes.

### miR–9-5p triggers the switch to glycolysis in trained immunity.

**Figure 4:**
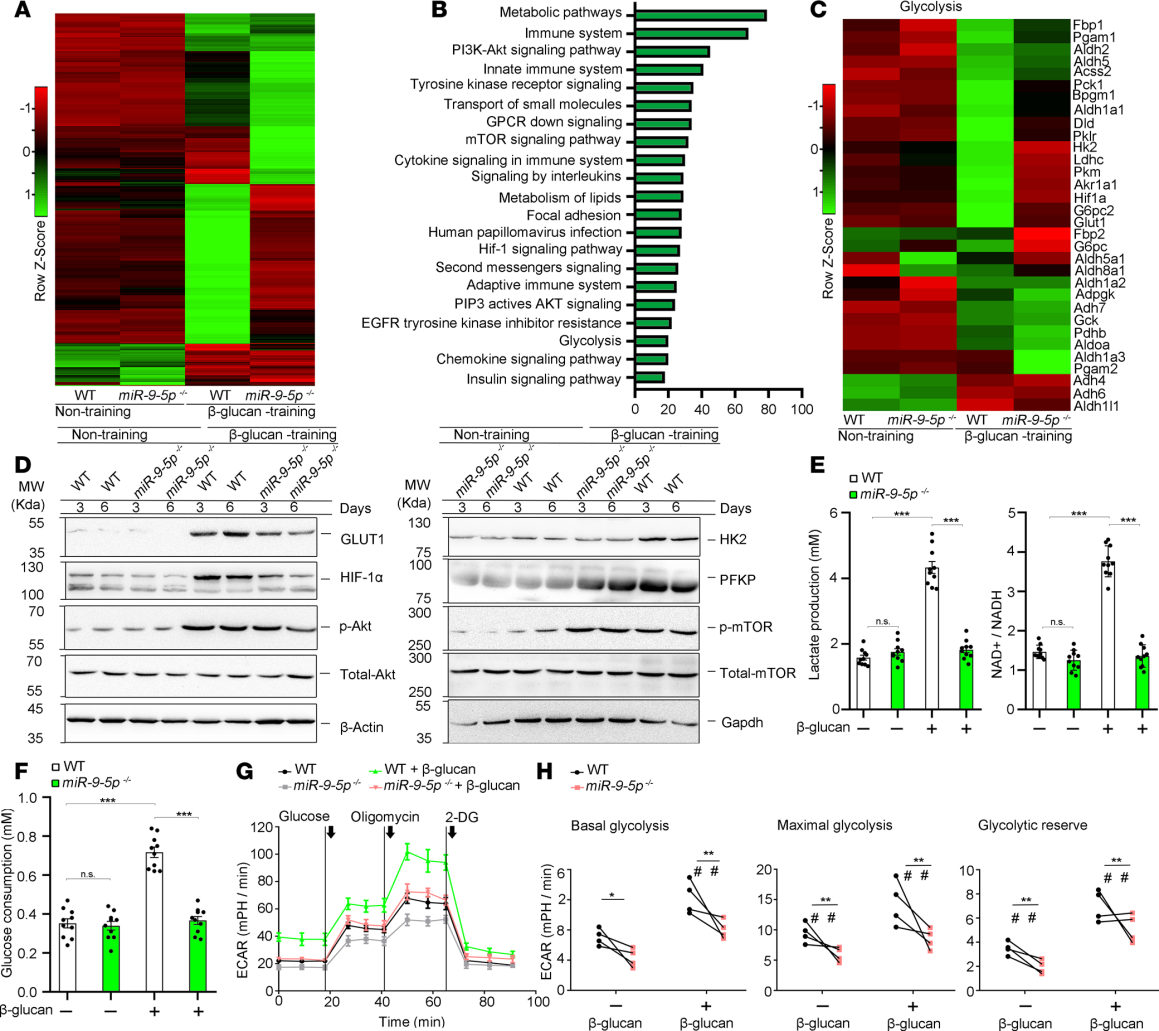
miR–9-5p triggers the switch to glycolysis in trained immunity. (**A**) Heatmap showed the relative mRNA expression in β-glucan–trained monocytes from WT or *miR–9-5p^–/–^* mice. The scale bar at the left ranges from red to green (low to high expression). RNA-seq was performed once in triplicate (*n* = 3). (**B**) KEGG enrichment analysis of up- or downregulated mRNAs in β-glucan–trained monocytes from WT or *miR–9-5p*^–/–^ mice. (**C**) Glycolysis pathway gene expression was determined according to **A**. (**D**) Western blot analysis examined p-Akt, Akt, p-mTOR, mTOR, GLUT1, HK2, HIF-1α, PFKP, and actin protein levels in β-glucan training of monocytes from WT or *miR–9-5p*^–/–^ mice (*n* = 3 independent experiments). (**E** and **F**) Dynamic changes in lactate production and NAD^+^/NADH (**E**), as well as glucose consumption (**F**) from day 3 of the β-glucan–trained monocytes from WT or *miR–9-5p*^–/–^ mice (*n* = 5 independent experiments). (**G** and **H**) Extracellular acidification rate (ECAR) after a glycolysis stress test upon sequential addition of glucose, oligomycin, and 2-deoxyglucose (2-DG), as indicated in β-glucan–trained monocytes from WT or miR–9-5p^–/–^ mice (**G**). Basal glycolysis, maximal glycolysis, and glycolytic reserve (**H**) (*n* = 4 independent experiments). In **E**, **F**, and **H**, data represent means ± SEM. ***P < 0.001 by 1-way ANOVA/Tukey’s multiple comparisons (**E** and **F**); dots show the individual data. *P < 0.05, **P < 0.01 by 2-tailed Student’s *t* test comparing WT and *miR–9-5p^–/–^*; ^#^*P <* 0.05 by paired Student’s *t* test comparing stimulated or not with β-glucan within the same genotype (**H**). Unprocessed original scans of blots are shown in Supplemental Figure 11.

To determine the molecular mechanism that miR–9-5p uses to induce trained immunity, we performed an unbiased assessment of whole-genome mRNA expression patterns after training WT and *miR–9-5p^–/–^* monocytes with β-glucan. Monocytes were trained as described above, and after 5 days of rest (before restimulation) (Figure 2E), monocyte-derived-macrophages were harvested for RNA sequencing (RNA-seq) analysis (Supplemental Table 2). Heatmap analysis revealed distinct expression patterns of some mRNAs when β-glucan–trained *miR–9-5p^–/–^* monocytes were compared with control samples (Figure 4A). Pathway analysis of these differentially regulated genes in β-glucan–trained monocytes between WT and *miR–9-5p^–/–^* mice showed that several pathways were involved in trained immunity, such as Akt/mTOR signaling, HIF-1α signaling, and inflammatory cytokine signaling (Figure 4B and Supplemental Figure 5A). Furthermore, the decreased glycolysis induction and increased oxidative phosphorylation were observed in *miR–9-5p^–/–^* monocytes compared with that of naive cells (Figure 4C and Supplemental Figure 5A). Monocytes trained with β-glucan were shown to switch from oxidative metabolism to glycolysis in an Akt/mTOR pathway–dependent manner (6). Thus, we hypothesized that miR–9-5p was involved in aerobic glycolysis reprogramming during β-glucan–induced trained immunity. Regarding the molecular pathway, Akt and mTOR were phosphorylated in response to β-glucan training in WT monocytes, being consistent with previous results (Supplemental Figure 2, A and C) but comparable between WT and *miR–9-5p^–/–^* monocytes (Figure 4D and Supplemental Figure 5B). In an analysis of glycolysis key proteins, hexokinase 2 (HK2) and glucose transporter-1 (GLUT1) were decreased upon β-glucan treatment in *miR–9-5p^–/–^* monocytes (Figure 4D), consistent with the qPCR results, in which HK2 and GLUT1 displayed similar reduction (Supplemental Figure 5C). Additionally, glucose consumption, lactate production, and the NAD^+^/NADH ratio were significantly reduced in *miR–9-5p^–/–^* monocytes induced with β-glucan compared with that in WT cells (Figure 4, E and F). Furthermore, we examined the extracellular acidification rate (ECAR) by glycolysis stress test in β-glucan–trained monocytes before LPS stimulation (Figure 4G). Training with β-glucan for 6 days increased ECAR in WT monocytes due to a metabolic shift; however, it decreased ECAR in *miR–9-5p^–/–^* monocytes (Figure 4G), as reflected by inhibited basal and maximal glycolysis, together with a lower glycolytic reserve (Figure 4H). In contrast, basal oxygen consumption after β-glucan training was increased in *miR–9-5p^–/–^* monocytes by approximately 50% compared with that in control monocytes (Supplemental Figure 5D). These data demonstrate that miR–9-5p switches the energy metabolism from oxidative phosphorylation to glycolysis in β-glucan–triggered trained immunity.

### miR–9-5p regulates the molecular, metabolic, and epigenetic hallmarks of trained immunity.

**Figure 5:**
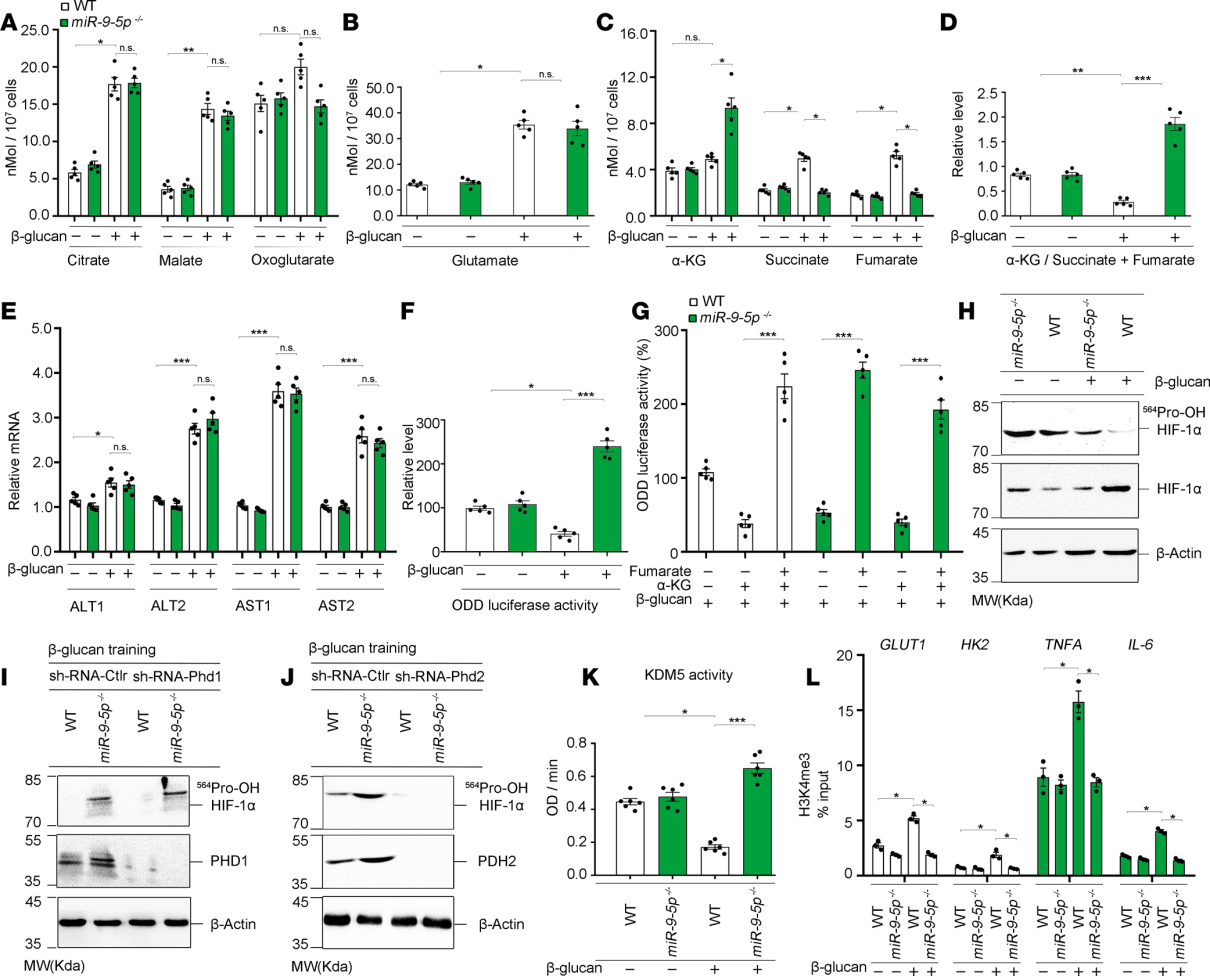
miR–9-5p regulates the molecular, metabolic, and epigenetic hallmarks of trained immunity. (**A**–**D**) Accumulation of citrate, malate, and oxoglutarate (**A**); glutamate (**B**); total content of α-KG, succinate, and fumarate (**C**); and the ratio of α-KG to succinate and fumarate (**D**) were determined using the cell lysates on day 6 in β-glucan versus nontrained monocytes from WT or *miR–9-5p^–/–^* mice (*n* = 3 independent experiments). (**E**) Enzymes involved in glutamate conversion into α-KG were screened using qPCR (*n* = 5 independent experiments). (**F**) ODD luciferase activity was measured in β-glucan–trained versus nontrained monocytes from WT or *miR–9-5p*^–/–^ mice (*n* = 5 independent experiments). (**G**) ODD luciferase activity was measured in monocytes treated with 100 μM α-KG, 100 μM fumarate, or mixtures of α-KG (100 μM) with 100 μM fumarate under training condition; the values were normalized to control (*n* = 5 independent experiments). (**H**) The hydroxylation level of HIF-1α was detected in β-glucan–trained monocytes from WT or *miR–9-5p*^–/–^ mice, using an antibody against hydroxylated HIF-1α at proline 564 (*n* = 3 independent experiments). (**I** and **J**) PHD1, but not PHD2, regulates HIF-1α stability (*n* = 3 independent experiments). (**K**) KDM5 activity was determined in β-glucan–trained monocytes from WT or *miR–9-5p^–/–^* mice (*n* = 6 independent experiments). (**L**) H3K4me3 level was determined at the promoter sites of *HK2*, *GLUT1*, *TNFA*, and *IL6* (*n* = 3 independent experiments). In **A**–**G**, **K**, and **L**, data represent means ± SEM. *P < 0.05, **P < 0.01, or ***P < 0.001 by 1-way ANOVA/Tukey’s multiple comparisons test (**A**–**F**, **K**, and **L**); ***P < 0.001 by 2-tailed Student’s *t* test (**G**). Unprocessed original scans of blots are shown in Supplemental Figure 11.

Induction of trained immunity is mediated via metabolites of the TCA cycle, including succinate, fumarate, and glutamate, which integrate immune and metabolic circuits to induce monocyte epigenetic reprogramming (18). We further examined whether these metabolic products were differentially accumulated in between WT and *miR–9-5p^–/–^* monocytes under β-glucan training. No change in citrate, malate, oxoglutarate, or glutamate was observed in *miR–9-5p^–/–^* and WT monocytes trained with β-glucan (Figure 5, A and B). Succinate and fumarate levels decreased approximately 2-fold in *miR–9-5p^–/–^* monocytes trained with β-glucan compared with that in WT cells; in contrast, α-KG level was increased (Figure 5C), resulting in the increased ratio of α-KG to succinate and fumarate (Figure 5D). Because α-KG is also derived from glutamate, we examined the expression of enzymes converting glutamate to α-KG, including glutamic pyruvate transaminase (ALT) and aspartate aminotransferase (AST), using qPCR. The mRNA expression of ALTs and ASTs were unchanged in both *miR–9-5p^–/–^* monocytes and WT cells trained with β-glucan (Figure 5E), suggesting that the elevation of α-KG was likely due to the increased conversion of ICT to α-KG. Proline hydroxylase (PHD), a member of the dioxygenase family, requires oxygen and α-KG for its activation. To further confirm that miR–9-5p loss elevated the level of α-KG, an oxygen-dependent degradation (ODD) domain luciferase assay was used to measure the PHD activity in WT and *miR–9-5p^–/–^* monocytes trained with β-glucan. We found that miR–9-5p deficiency reduced ODD luciferase activity under β-glucan training (Figure 5F); however, addition of fumarate reversed the increase of ODD activity in *miR–9-5p^–/–^* monocytes (Figure 5G). The results demonstrated that miR–9-5p deficiency increased the ratio of α-KG to succinate and fumarate in β-glucan–trained immunity. α-KG regulates HIF-1α stabilization by promoting hydroxylation and, therefore, destabilizing HIF-1α, which mounts trained immunity (7). We measured the hydroxylation of HIF-1α (P564), and we found that miR–9-5p deficiency in β-glucan–trained monocytes increased HIF-1α hydroxylation (Figure 5H). HIF-1α is directly hydroxylated at prolines 564 via PHD, leading to the binding of E3 ligase VHL and consequent ubiquitination and degradation of HIF-1α (19). Knockdown of PHD2, but not PHD1, significantly decreased HIF-1α hydroxylation in β-glucan training of *miR–9-5p^–/–^* monocytes (Figure 5, I and J). These results indicated that miR–9-5p contributes to the suppression of HIF-1α hydroxylation in β-glucan–trained immunity.

β-Glucan training of monocytes resulted in decreased biological activity of KDM5 demethylases, which corresponds to the time point with the increasing fumarate concentration (7). α-KG is a known cofactor of the KDM5, which can actively remove lysine trimethylation of H3K4me3 (20). KDM5 activity could be inhibited by fumarate but could be restored by the addition of α-KG (Supplemental Figure 6A). Due to the elevated ratio of α-KG/succinate and fumarate, we assessed whether miR–9-5p loss affected the KDM5 activity in β-glucan–trained monocytes. We found that KDM5 activity was significantly increased in β-glucan training of *miR–9-5p^–/–^* monocytes compared with that in WT cells (Figure 5K). Addition of fumarate to *miR–9-5p^–/–^* monocytes reduced the KDM5 activity to the level of WT cells (Supplemental Figure 6, B and C). H3K4me3 modification at the promoters of proinflammatory cytokine and aerobic glycolysis genes was specifically induced by β–glucan training (7). Epigenetic changes in H3K4me3 at the promoter sites of *TNFA*, *IL-6*, *HK2*, and *GLUT1* were abolished in β-glucan training of *miR–9-5p^–/–^* monocytes (Figure 5L). Fumarate addition restored the H3K4me3 levels at *TNFA* promoters in *miR–9-5p^–/–^* monocytes trained by β-glucan (Supplemental Figure 6, D–F). Therefore, miR–9-5p controls the ratio of α-KG/fumarate, resulting in HIF-1α stability and epigenetic changes induced by β-glucan training.

To further confirm whether miR–9-5p indeed regulates the immunometabolic and epigenetic changes of trained immunity, *miR–9-5^–/–^* monocytes were transfected with or without a miR–9-5p rescued plasmid; then, the immunity-associated cytokines and metabolic products induced by β-glucan were analyzed. We observed that compensatory expression of miR–9-5p rescued the glucose consumption, glycolysis, α-KG, and fumarate content induced by β-glucan in *miR–9-5^–/–^* monocytes (Supplemental Figure 7, A–D). Additionally, H3K4me3 was recurrently enriched at the *TNFA* promoter in *miR–9-5^–/–^* monocytes (Supplemental Figure 7, E and F), concurring with final cytokines production. Thus, miR–9-5p regulates the molecular, metabolic, and epigenetic hallmarks of trained immunity.

### miR–9-5p regulates the expression of IDH3α.

**Figure 6:**
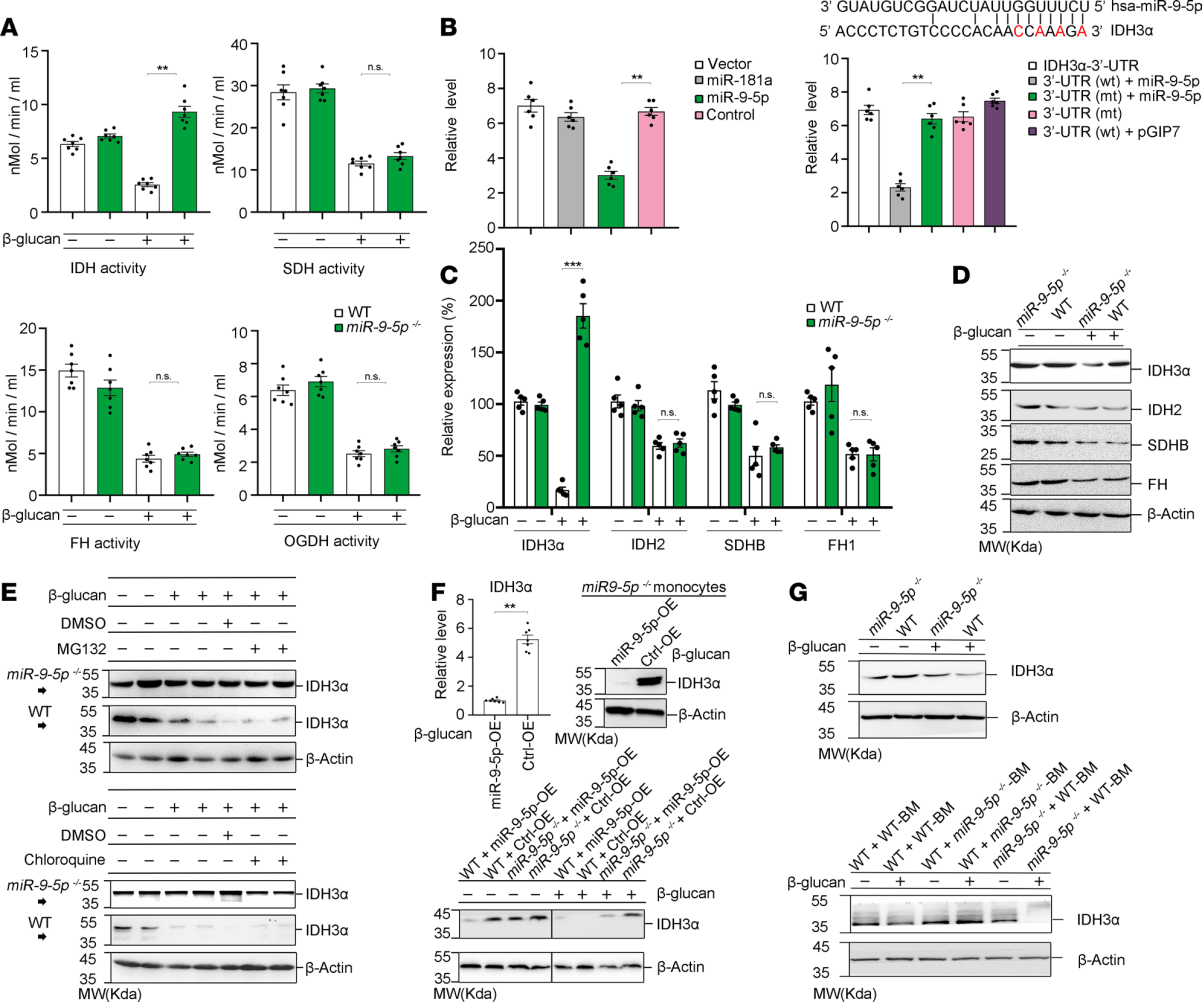
miR–9-5p regulates the expression of IDH3α. (**A**) Activity of IDH, SDH, FH, and OGDH in β-glucan–trained monocytes from WT or *miR–9-5p*^–/–^ mice (*n* = 3 independent experiments). (**B**) miR–9-5p regulates IDH3α expression. Left: luciferase activity of IDH3α 3′UTR. Right: luciferase activity in WT or mutant IDH3α 3′UTR groups. The nucleotides indicated in red for the IDH3α seed sequence were mutated to complementary nucleotides (*n* = 3 independent experiments). (**C**) qPCR analysis of IDH3α, IDH2, SDHB, and FH1 in β-glucan–trained monocytes from WT or *miR–9-5p*^–/–^ mice (*n* = 3 independent experiments). (**D**) Western blot analysis of IDH3α, FH, SDHB, and IDH2 expression in β-glucan–trained monocytes from WT or *miR–9-5p*^–/–^ mice (*n* = 3 independent experiments). (**E**) Western blot analysis of IDH3α expression in β-glucan–trained monocytes from WT or *miR–9-5p*^–/–^ mice in the presence of 10 μM MG132 (proteasome inhibitor) or 50 μM chloroquine (lysosome inhibitor) (*n* = 3 independent experiments). (**F**) IDH3α expression in monocytes from WT or *miR–9-5p*^–/–^ mice transfected with miR–9-5p overexpression or a control under β-glucan training (*n* = 3 independent experiments). (**G**) IDH3α expression determined by Western blot in monocytes from trained radiation chimeras and WT and *miR–9-5p^–/–^* mice, according to Figure 3E (*n* = 3 independent experiments). Data represent means ± SEM. **P < 0.01, ***P < 0.001 by 2-tailed Student’s *t* test (**A**, **C**, and **F**); **P < 0.01 by 1-way ANOVA/Tukey’s multiple comparisons test (**B**). Unprocessed original scans of blots are shown in Supplemental Figure 11.

Dysregulation of genes encoding the Kreb’s cycle enzymes succinate dehydrogenase (SDH), fumarate hydratase (FH), and IDH results in the accumulation of succinate and fumarate and a reduced ratio of α-KG to succinate and fumarate (21). Here, the activity of IDH, SDH, and FH was significantly decreased in β-glucan training of monocytes. IDH activity in *miR–9-5p^–/–^* monocytes was reversed to the level of WT cells, whereas SDH and FH activation changed little (Figure 6A). RNA-seq data showe that IDH3α expression was increased under β-glucan training conditions in *miR–9-5p^–/–^* monocytes compared with that in WT; in contrast, the levels of IDH2, SDHB, and FH1 were unchanged (Supplemental Figure 8A). Thus, we inferred that miR–9-5p regulated the induction of trained immunity through IDH3α targeting. We found that miR–9-5p reduced firefly luciferase expression in the IDH3α 3′UTR luciferase reporter assay (Figure 6B). When 4 nucleotides in the IDH3α 3′UTR that were predicted to associate with miR–9-5p were mutated, miR–9-5p lost the ability to inhibit firefly luciferase expression, suggesting that miR–9-5p was involved in the downregulation of IDH3α (Figure 6B). The expression of IDH3α was increased in β-glucan training of *miR–9-5p^–/–^* monocytes compared with WT cells (Figure 6, C and D). Similarly, miR–9-5p inhibition promoted the level of IDH3α in β-glucan training of human monocytes (Supplemental Figure 8, B and C). No change in IDH3α expression was found between naive cells and miR–9-5p–deficient monocytes after treatment with MG132 or chloroquine (Figure 6E and Supplemental Figure 8D), suggesting that downregulation of IDH3α occurred mainly through transcriptional regulation. To further confirm that miR–9-5p inhibited IDH3α expression, *miR–9-5p^–/–^* monocytes were transfected with miR–9-5p or a scrambled control (Figure 6F, top). Rescue of miR–9-5p expression reduced IDH3α levels in *miR–9-5p^–/–^* monocytes upon exposure to β-glucan (Figure 6F and Supplemental Figure 8, E and F). Moreover, the expression of IDH3α was reduced in monocytes from trained WT + WT-BM and *miR–9-5p^–/–^* + WT-BM mice versus *miR–9-5p^–/–^* mice and WT *+ miR–9-5p^–/–^*-BM chimeras (Figure 6G). These results firmly establish an essential role of miR–9-5p in trained immunity via targeting IDH3α.

### miR–9-5p regulates DNA hypomethylation.

IDH3α loss of function affects DNA methylation (10). Having identified IDH3α as a target of miR–9-5p, we evaluated whether and to what extent differential methylation in human monocytes transfected with miR–9-5p and a scrambled control. RNA-seq analysis identified 3057 differentially expressed genes (Supplemental Figure 9A). The higher number of hypomethylated relative to hypermethylated CpGs revealed an overall global increase in DNA hypomethylation in miR–9-5p–overexpressing monocytes (Supplemental Figure 9B). Integrative analysis of gene expression and methylation data show that expression of 1189 genes (out of 3057) correlated with their CpG hypomethylation status (Supplemental Figure 9C). Pathway enrichment analysis of genes with correlation between gene expression and demethylation identified cellular metabolic pathway and inflammatory response as key pathways regulated through hypomethylation-driven expression changes upon miR–9-5p overexpression (Supplemental Figure 9D). Correlation for CpG sites was mainly located within island and shores, compared with shelf and open sea (Supplemental Figure 9E). The promoter regions of *HK2* and *TNFA* showed hypomethylation, while *IDH3A* methylated status changed little (Supplemental Figure 9F).

Additionally, knocking down miR–9-5p partially resulted in DNA hypomethylation of the *IDH3A* promoter under training conditions (Supplemental Figure 9, G and H). Together, these results indicate that the miR–9-5p–controlled immunometabolic change in β-glucan–trained monocytes may occur through hypomethylation of trained immune genes.

### IDH3α impairs the metabolic and epigenetic changes in β-glucan–trained immunity.

**Figure 7:**
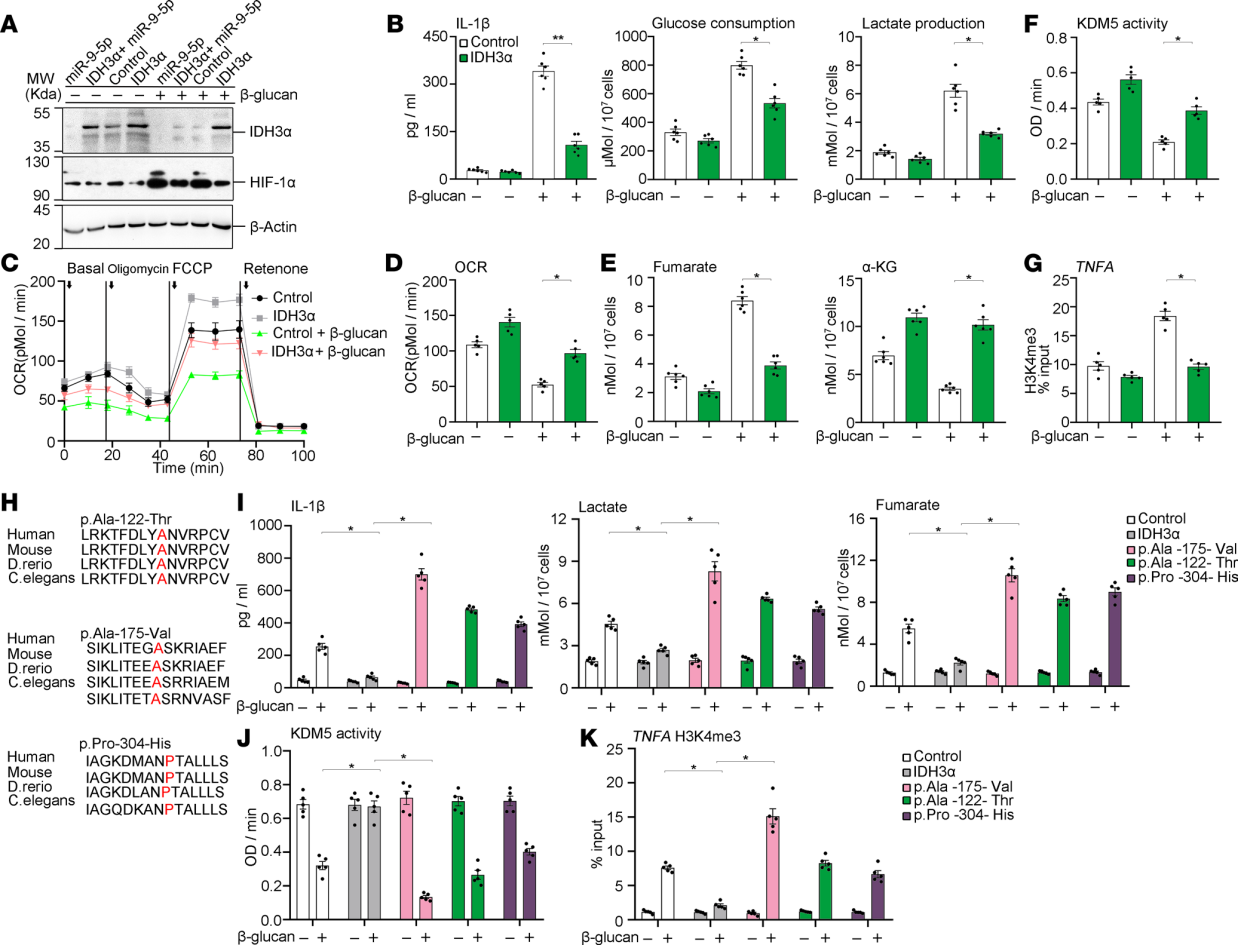
IDH3α impairs the metabolic and epigenetic changes in β-glucan–trained immunity. (**A**) miR–9-5p inhibits the expression of IDH3α in β-glucan–trained monocytes overexpressing IDH3α (*n* = 3 independent experiments). (**B**) Analysis of IL-1β, lactate production, and glucose consumption in β-glucan–trained monocytes overexpressing IDH3α (*n* = 5 independent experiments). (**C** and **D**) Basal oxygen consumption, oxidative phosphorylation, and maximum respiration capacity of β-glucan–trained monocytes overexpressing IDH3α (**C**). The histogram displays the basal level of oxygen consumption (**D**) (*n* = 5 independent experiments). (**E**–**G**) Relative α-KG and fumarate content (**E**), KDM5 activity (**F**), and H3K4me3 level at the promoter sites of *TNFA* (**G**) in β-glucan–trained monocytes with or without expression of IDH3α (*n* = 5 independent experiments). (**H**) Multiple sequence alignment of IDH3α orthologs around missense variant sites (highlighted in red) generated using NCBI HomoloGene. (**I**–**K**) IL-1β level, lactate production, and glucose consumption (**I**); KDM5 activity (**J**); and H3K4me3 level at promoter sites of *TNFA* (**K**) in β-glucan–trained monocytes with or without IDH3α mutant (*n* = 5 independent experiments). Data are shown as means ± SEM. *P < 0.05, **P < 0.01 by 2-tailed Student’s *t* test (**B**, **D**, **E**, **F**, and **G**); *P < 0.05 by 1-way ANOVA/Tukey’s multiple comparisons test (**I**, **J**, and **K**). Unprocessed original scans of blots are shown in Supplemental Figure 11.

To examine whether IDH3α affected the induction of trained immunity via β-glucan, we generated IDH3α-overexpressing monocytes (Figure 7A). We found that IDH3α gain of function inhibited IL-1β production, glucose consumption, and lactate production in β-glucan training of monocytes (Figure 7B). Downregulation of oxygen consumption upon exposure to β-glucan was partially restored in IDH3α-overexpressing monocytes (Figure 7, C and D). The α-KG level was increased approximately 2-fold in IDH3α-overexpressing monocytes trained with β-glucan; in contrast, fumarate level was decreased (Figure 7E). KDM5 activity was also greatly increased in monocytes overexpressing IDH3α (Figure 7F), together with the decrease of H3K4me3 level at *TNFA* promoter (Figure 7G). Of note, β-glucan–trained monocytes with an IDH3α mutation showed increased production of IL-1β, lactate, and fumarate compared with that in IDH3α-overexpressing cells (Figure 7, H and I). Moreover, IDH3α-mutant monocytes showed the decreased KDM5 activity and the increased H3K4me3 enrichment at the *TNFA* promoter (Figure 7, J and K). Thus, IDH3α dampened the energy metabolism and epigenetic changes in monocytes trained with β-glucan.

### Monocytes with an IDH3α mutation (p.Ala-175-Val) from patients with autosomal recessive retinitis pigmentosa (ArRP) show a trained immunity phenotype.

**Figure 8:**
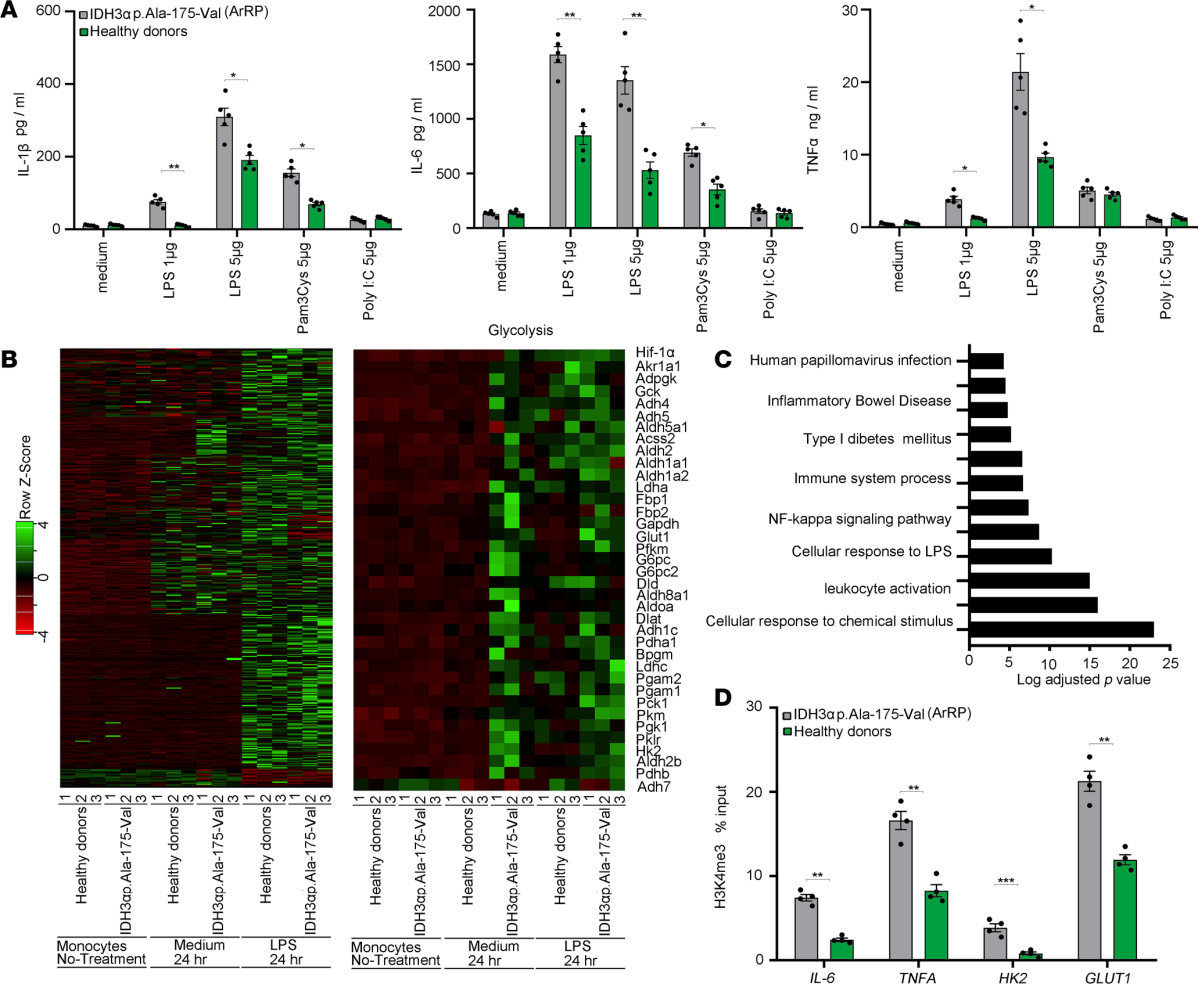
Monocytes with an IDH3α mutation (p.Ala-175-Val) from patients with autosomal recessive retinitis pigmentosa (ArRP) show a trained immunity phenotype. (**A**) Monocytes from patients with autosomal recessive retinitis pigmentosa (ArRP), who had the IDH3α (p.Ala-175-Val) mutation, and matching healthy volunteers were exposed ex vivo to several ligands for 24 hours. Cytokine expression levels were measured in the supernatants (*n* = 5 independent experiments). (**B**) Heatmap of individual donors for genes that show LPS responses in controls and patients with ArRP who had IDH3α (p.Ala-175-Val) mutation. Monocytes were either untreated or stimulated for 24 hours with media or LPS. RNA-seq was performed once in triplicate (*n* = 3 versus 3). (**C**) Pathway associated with genes that show higher expression in ArRP patients monocytes exposed to 24-hour RPMI compared with controls (*n* = 3 versus 3). (**D**) H3K4me3 levels were determined at the promoter sites of *TNFA*, *IL-6*, *HK2*, and *GLUT1* in ArRP patient monocytes exposed to 24-hour RPMI compared with controls (*n* = 4 independent experiments). Data are shown as means ± SEM, *P < 0.05, **P < 0.01, or ***P < 0.001 by 2-tailed Student’s *t* test (**A** and **D**).

IDH3α variants were identified as crucial causes of typical ArRP (22). Without stimulation, ArRP patients suffer sustained inflammation attacks, characterized by increased macrophage activity and increased chemokine and cytokine levels (23). However, the molecular mechanism of this increased inflammatory state is still unclear. We hypothesized that this is due to a trained-like phenotype of ArRP monocyte–derived macrophages with IDH3α mutants, which likely induces the critical glycolytic switch and the epigenetic change. We analyzed monocytes from 3 adult patients with ArRP that had an IDH3α mutation (p.Ala-175-Val). In line with our hypothesis, monocytes from these patients produced significantly more TNF-α, IL-6, and IL-1β upon stimulation with several ligands compared with healthy controls (Figure 8A). RNA-seq analysis revealed that IDH3α (p.Ala-175-Val) variant monocytes exposed to media or LPS for 24 hours, or left unstimulated (Supplemental Table 3), showed differential expression of multiple genes (Figure 8B). Pathway analysis of the these expression data is displayed in Figure 8C and shows upregulated inflammatory pathways, such as NF-κB signaling, supporting the hypothesis of a hyperinflammatory state. Although the expression profiles of Akt-mTOR pathway showed little change (Supplemental Figure 10A), we observed increased expression of glycolysis and cytokine genes in ArRP patient monocytes after 24 hours of media treatment, as well as decreased oxidative phosphorylation pathway activity (Figure 8B and Supplemental Figure 10A). Glucose consumption and lactate production were increased in monocytes with an IDH3α mutation (p.Ala-175-Val), compared with that in healthy donor cells, whereas the ratio of α-KG to fumarate was decreased (Supplemental Figure 10, B and C). Moreover, KDM5 activity was decreased in IDH3α-mutated monocytes (Supplemental Figure 10D). H3K4me3 levels at *TNFA*, *IL-6*, *HK2*, and *GLUT1* promoters were higher in ArRP monocytes with an IDH3α mutation than that in normal cells (Figure 8D). These results demonstrate that IDH3α loss of function may be one of the main contributors to the inflammatory signatures in ArRP.

## Discussion

Trained immunity describes the ability of innate immune cells to form immunological memories of prior encounters with pathogens. Recollection of these memories during a secondary encounter manifests a broadly enhanced inflammatory response characterized by the increased transcription of innate immune genes (3). Despite this phenomenon having been described over a decade ago, our understanding of the molecular mechanisms responsible for this phenotype is still incomplete. Herein, β-glucan–trained *miR–9-5p^–/–^* monocytes showed decreased IL-1β, IL-6, and TNF-α production after LPS stimulation. Trained *miR–9-5p^–/–^* mice produced decreased proinflammatory cytokines upon rechallenge in vivo and had worse protection against *C*. *albicans* infection. We highlight the mechanistic role of miR–9-5p in the establishment and maintenance of discrete, long-lasting epigenetic modifications that are causal to the trained immune response. The discovery of the central role of miR–9-5p function in the establishment of trained immunity has revealed a class of therapeutic targets for potentially controlling inflammatory responses in a discrete manner. Furthermore, as the catalog of functionally investigated microRNAs transcripts grow, we foresee that more microRNAs that are important for the regulation of trained immunity and inflammation will be revealed.

Trained immunity undergoes an oxygen-independent metabolic switch from oxidative phosphorylation to aerobic glycolysis (24). However, it is not clear what regulates this metabolic reprogramming. Previously, the Akt/mTOR pathway was shown to participate in the immunometabolic changes in trained immunity (6). In this context, we found that pharmacological inhibition or downregulation of the Akt/mTOR pathway reduced miR–9-5p expression. To further elucidate the mechanisms through which Akt/mTOR regulates miR–9-5p level, we turned to GSK3β, which binds directly to the microprocessor complex and facilitates microRNA biogenesis (25). Additionally, Akt/mTOR inhibition reduced GSK3β protein levels, and the increase in microRNA biogenesis associated with Akt/mTOR activation was reversed by GSK3β inhibition (14), suggesting that Akt/mTOR regulates microRNA biogenesis via GSK3β. In this context, we found that total GSK3β level was increased in monocytes trained with β-glucan. Also loss of function of Akt/mTOR led to reduction of GSK3β. GSK3β inhibition also decreased miR–9-5p level in β-glucan–trained monocytes. These results suggest that β-glucan–trained immunity could induce miR–9-5p expression via activation of the Akt/mTOR/GSK3β pathway. *miR–9-5p^–/–^* monocytes trained with β-glucan did not alter the phosphorylation of Akt and mTOR, and they supported miR–9-5p acting potentially downstream to it. Upon β-glucan training, HIF-1α plays an important role in mounting glycolysis via Akt/mTOR activation (26). Expression of HIF-1α at protein levels was significantly reduced in β-glucan–trained *miR–9-5p^–/–^* monocytes compared with WT cells. Actually, miR–9-5p deficiency promoted the PHD2-mediated hydroxylation of HIF-1α, indicating that miR–9-5p stabilized HIF-1α in trained immunity. We also found that, in *miR–9-5p^–/–^* monocytes, lactate production and glucose consumption were decreased, whereas oxygen consumption was increased, demonstrating that induction of miR–9-5p directly promoted the switch of glucose metabolism from oxidative phosphorylation to glycolysis. Our results identify miR–9-5p as a critical molecular switch promoting aerobic glycolysis.

Genome-wide changes in histone modifications have been shown to underlie trained immunity in monocytes (27). Trained immunity induces the accumulation of key metabolites in the TCA cycle, such as fumarate and succinate, and decreased the level of α-KG (28, 29). The metabolites act as cofactors for epigenetic enzymes, resulting in the induction of histone modifications, such as H3K4me3, by regulating KDM5 activity (8). However, the molecular mechanisms underlying the metabolites accumulation in trained immunity have not been deciphered. Here, miR–9-5p deficiency increased the level of effective α-KG by promoting the ratio of α-KG to fumarate and succinate. α-KG acts as a cofactor to enhance KDM5 activity for H3K4 demethylation, whereas fumarate, having a similar molecular structure to α-KG, can act as an antagonizing factor (7). We found that KDM5 activity was increased in β-glucan–trained *miR–9-5p^–/–^* monocytes, and the trimethylation of H3K4 was reduced at the promoters of *TNFA*, *IL-6*, *HK2,* and *GLUT1* due to the increase in the amount of α-KG. This indicated that the metabolic and epigenetic changes were intertwined and highly dependent on miR–9-5p expression in trained immunity. Diminished IDH3 activity results in the metabolic switch from oxidative phosphorylation to glycolysis (30). IDH3α inactivation reduced the ratio of α-KG to fumarate and succinate (10). In this context, IDH3α inhibited the metabolic switch from oxidative phosphorylation to glycolysis and increased the α-KG/fumarate ratio, impairing epigenetic changes in trained monocytes by increasing KDM5 activity. IDH3α gain of function reduced the enrichment of H3K4me3 at the promoters of *TNFA* in monocytes upon β-glucan training. Actually, miR–9-5p directly targeted IDH3α in the processes of trained immunity. Recently, IDH3α was found to interact with cytosolic serine hydroxymethyltransferase (cSHMT) to enhance *S*-adenosyl methionine (SAM) generation (10), which involves depositing the repressive H3K9me3 mark in trained immunity (31). Gain or loss of IDH3α function regulates the expression of the methyl group donor SAM, which should be explored in β-glucan–trained immunity. It is likely that IDH3α downregulation led to the immunometabolic programs, contributing to the induction of β-glucan–trained immunity. Thus, our study revealed that the miR–9-5p/IDH3α axis is one of the main contributors to the immunometabolic and epigenetic remodeling in trained immunity.

ArRP is an inherited form of retinal dystrophy caused by inherited or acquired mutations in over 50 different genes, including *IDH3A* (22, 32). Loss-of-function and missense mutations in IDH3α are implicated in families exhibiting ArRP (22). In this disease, there is increased macrophage activity and levels of chemokines and cytokines (IL-1β) in patients and rodent models (23, 33). However, it is unclear whether this increased inflammatory state is the cause or a consequence of this currently untreatable disease. We found that an IDH3α mutation led to the increased production of proinflammatory cytokines and accumulation of TCA cycle metabolites in β-glucan–induced trained immunity. In addition to the beneficial effects of trained immunity as a host defense mechanism, trained immunity plays a deleterious role in the induction and/or persistence of inflammatory diseases if inappropriately activated (34, 35). Thus, we hypothesized that induction of the trained immunity phenotype, characterized by exaggerated cytokine production from monocytes due to an IDH3α mutation, may play a role in the pathophysiology of ArRP. This hypothesis was validated via the demonstration of excessive cytokine production in ArRP monocytes with an IDH3α mutation (p.Ala-175-Val), accompanied with transcriptional and epigenetic changes. In previous studies, NLRP3 expression was significantly upregulated in the early-onset model up to 7–16 weeks, and IL-1β expression was still upregulated in the late-onset model from 16 weeks (33, 36), suggesting that, in addition to the mechanism dependent on NLRP3 inflammasome activation, an IDH3α mutation may play a causative role in the development of the hyperinflammatory state observed in ArRP. However, the study was limited by the small sample size, and more samples from different populations are needed to confirm this hypothesis. Because the loss of function of *IDH3A* in mice is lethal, further studies are required to investigate the role of IDH3α in trained immunity via the generation of *IDH3A* homozygous mutation in mice to reveal the link between the inflammatory state induced by IDH3α and ArRP. We have provided an insight into understanding the hyperinflammatory state of patients with ArRP regarding trained immunity.

In conclusion, miR–9-5p promoted the rewiring of cellular metabolism that modulates the epigenetic programming of metabolic genes in β-glucan training of monocyte. As a direct target of miR–9-5p, IDH3α impaired the key metabolite changes (a switch from oxidative phosphorylation to aerobic glycolysis and the accumulation of fumarate and succinate). Moreover, monocytes from patients with ArRP, having an IDH3α mutation, may acquire a trained immunity phenotype, characterized by the increased expression of cytokines and genes involved in the glycolysis pathway. We have identified the miR–9-5p/IDH3α axis as a crucial mechanism linking the stimulation of innate immune pathways with the induction of epigenetic and metabolic changes in trained immunity. Thus, therapeutic strategies targeting the miR–9-5p/IDH3α axis may represent a promising approach for prevention of inflammation.

## Methods

### Mice and human samples.

*miR–9-5p^–/–^* mice were generated through the CRISPR/Cas9 method (37). In vitro–synthesized Cas9 mRNA and gRNA were comicroinjected into the C57BL/6 zygotes. The 3 gRNA sequences used to generate the KO mice were 5′-GCTGGTTAAAGAGGAAGAAG NGG-3′, 5′GCTGATTCAAAGATCTGCTC NGG3′, and 5′GGCTTTATACATCTCAGTAG NGG 3′. C57BL/6 mice were obtained from HLK Bio Co. Ltd. All mice were bred in specific pathogen–free conditions at the Laboratory Animal Center of Fudan University. Female and male mice, 6–8 weeks old, were used.

Buffy coats from healthy volunteers and ArRP patients obtained from the the Sixth Affiliated Hospital of Guangzhou Medical University were ethically approved by the Institutional Human Ethics Committee.

### Trained immunity in vitro models.

Blood mononuclear cells were isolated from WT and *miR–9-5p*^–/–^ mice using Ficoll-Paque (GE Healthcare) as previously described (7). Briefly, 1 × 10^7^ to 2 × 10^7^ PBMCs were layered on top of a hyperosmotic Percoll solution (48.5% Percoll [Sigma-Aldrich], 41.5% sterile H_2_O, and 0.16 M filter-sterilized NaCl) and centrifuged for 15 minutes at 600*g* at room temperature. The interphase layer was isolated, and cells were washed with cold PBS. Human monocytes were purified by MACS depletion of CD3^+^, CD19^+^, and CD56^+^ cells from the PBMCs: CD3 MicroBeads (catalog 130-050-101), CD19 MicroBeads (catalog 130-050-301), and CD56 were purchased from Miltenyi Biotec (catalog 130-050-401) and used according to the manufacturer’s protocol. The efficacy of depletion was controlled by flow cytometry (B53000, Beckman-Coulter) and was higher than 95%. Cells were resuspended in RPMI culture medium (RPMI medium, Invitrogen) supplemented with 10 μg/mL gentamicin, 10 mM glutamax, and 10 mM pyruvate and counted.

Monocytes (1 × 10^6^) were plated in 96-well plates (200 μL final volume; Corning) and stimulated with Gibco RPMI 1640 medium or β-glucan (Invitrogen) at 5 μg/mL for 24 hours in the presence of recombinant GM-CSF at 20 ng/mL as previously described (38). Then, cells were washed and rested for 4 days in the culture medium with 10% FBS. On day 6, a final wash was performed, and cells were stimulated with medium or 1 μg/mL of LPS (Sigma-Aldrich) (39).

To measure IL-1β, TNF-α, and IL-6 expression, after 24 hours of LPS stimulation, supernatants were collected for ELISA. All ELISA kits were purchased from R&D Systems. When required, monocytes were preincubated for 30 minutes before β-glucan stimulation with 10 μM MG132 or 50 μM chloroquine (Sigma-Aldrich). miR–9-5p mimics or miR–9-5p inhibitor (RiboBio Co. Ltd) was also used at the indicated doses on β-glucan–trained BMDMs (toxic for nontrained cells). To test receptor expression and cell viability, we plated 1 × 10^6^ cells in nontreated 24-well plates (200 μL final volume; Corning) and followed the training scheme as described above. Cell viability, Dectin-1, TLR2, and TLR4 expressions were assessed on day 6 before LPS stimulation. Cells were collected in PBS/EDTA and stained with ice-cold FACS buffer for flow cytometry analysis. To test the role of the Akt/mTOR/GSK3β pathway in trained immunity, we added the specific inhibitors together with β-glucan for the first 24 hours in different doses as follows: DMSO (control), rapamycin (100 nM), metformin (30 mM), AICAR (200 nM), ascorbate (50 μM), AR-A014418 (5 μm), or MK2206 (10 μm) (Supplemental Table 4).

### Trained immunity in vivo models.

WT and *miR–9-5p*^–/–^ mice, 8–12 weeks old, were trained with either 2 i.p. injections of 1 mg of β-glucan particles on days –7 and –4, or with *C*. *albicans* at approximately 2 × 10^4^ cfu i.v. on day –7 (6). PBS was used as the control. Seven days later, mice were challenged with 10 μg of LPS i.p., and 3 hours later, blood was collected to assess serum TNF-α, IL-1β, and IL-6 levels. Alternatively, mice were lethally infected with 2 × 10^6^ to 3 × 10^6^ cfu of *C*. *albicans* (i.v.) and monitored daily for general health and survival, following institutional guidance. Data presented are the combined survival data (Kaplan-Meier) from 3 independent experiments. A log-rank test was used to assess the statistical significance between the groups. Kidney fungal burden at the indicated time points after infection was determined by plating organ homogenates obtained mechanically over 70 μm cell strainers after slicing the tissue, in serial dilutions on yeast extract peptone dextrose medium agar plates; CFUs were counted after growth at 30°C for 48 hours, and data were shown as CFUs in the total kidney.

### Radiation chimeras.

Details and the associated references are provided in the Supplemental Methods.

### Ch-IP analysis.

Details and the associated references are provided in the Supplemental Methods.

### Metabolic status analysis.

Details are provided in the Supplemental Methods.

### KDM5 activity assay.

Details are provided in the Supplemental Methods.

### DNA global methylation array.

Details and the associated references are provided in the Supplemental Methods.

### Integrative analysis of expression and methylation data.

Details and the associated references are provided in the Supplemental Methods.

### Statistics.

The statistical analysis was performed using Prism (GraphPad Software). Two-tailed Student’s *t* test was used for comparisons between 2 groups with a normal distribution, and the differences in means among multiple groups were analyzed using 1-way ANOVA for continuous variables with normal distribution. Comparison of survival curves was carried out using the log-rank (Mantel-Cox) test. For all experiments, *P <* 0.05 was considered statistically significant. Correlation analysis was performed based on Pearson’s coefficient. Different conditions within the same genotype in a particular experiment, although not connected by a matter of clarity, were also pair analyzed, and statistically significant differences are indicated by symbols. For all data in which 3 or more independent measurements are reported, data are displayed as mean ± SEM.

### Study approval.

Animal experiments were performed as per ethical guidelines approved by Fudan University’s IACUC. Buffy coats from healthy volunteers and ArRP patients obtained at the Sixth Affiliated Hospital of Guangzhou Medical University were ethically approved (approval no. GD2019-71) by the Institutional Human Ethics Committee of Guangzhou Medical University, Guangzhou, China.

## Author contributions

HS and YX contributed resources; HS, QG, ZL, and YX contributed data curation; HS, QG, and YX contributed software; HS and YX contributed formal analysis; HS, QG, JH, ZL, and YX independently repeated experiments and independently analyzed data; HS, QG, JH, S Weng, S Wu, XW, ZL, and YX contributed investigation; HS, JH, S Weng, S Wu, CS, XW, and YX contributed visualization; HS, JH, and YX contributed methodology; HS and YX contributed writing of the original draft; HS and YX contributed manuscript review and editing; QG and YX contributed funding acquisition; HS, QG, and YX contributed project administration; TZ, ZZ, and YZ contributed animal welfare; HS and YX contributed conceptualization; and YX contributed supervision. The order among co–first authors was determined by their contribution to performing experiments and writing the manuscript. HS is listed first, as he initiated the project.

## Supplementary Material

Supplemental data

Supplemental Table 1

Supplemental Table 2

Supplemental Table 3

## Version 1 05/10/2021

Electronic publication
