# miR–9-5p regulates immunometabolic and epigenetic pathways in **β**﻿﻿-glucan–trained immunity via IDH3**α**

**DOI:** 10.1172/jci.insight.159640

**Published:** 2022-03-22

**Authors:** Haibo Su, Zhongping Liang, ShuFeng Weng, Chaonan Sun, Jiaxin Huang, TianRan Zhang, Xialian Wang, Shanshan Wu, Zhi Zhang, Yiqi Zhang, Qing Gong, Ying Xu

Original citation: *JCI Insight*. 2021;6(9):e144260. https://doi.org/10.1172/jci.insight.144260

Citation for this corrigendum: *JCI Insight*. 2022;7(6):e159640. https://doi.org/10.1172/jci.insight.e159640

Information on data availability was omitted from Methods. The correct information is below.

Data availability

Raw data were deposited in the National Genomics Data Center’s Genome Sequence Archive (GSA CRA005771, CRA005786, HRA001843, and HRA001860).

The authors regret the error.

## Version 1 03/22/2022

Electronic publication

