# Supplementary material for: miR–9-5p regulates immunometabolic and epigenetic pathways in β-glucan–trained immunity via IDH3α
Source: JCI Insight. 2021 May 10;6(9):e144260. doi: 10.1172/jci.insight.144260 (PMC8262351; doi:10.1172/jci.insight.144260)
Supplement: Supplemental data [file jciinsight-6-144260-s192.pdf]

Supplemental Information

miR-9-5p Regulates Immunometabolic and Epigenetic Pathways in β-glucan-Trained Immunity via IDH3α

Haibo Su[1,2#], Zhongping Liang[3#], Shufeng Weng[2], Chaonan Sun[1], Jiaxin Huang[1], Tianran Zhang[2], Xialian Wang[1], Shanshan Wu[1], Zhi Zhang[1], Yiqi Zhang[1], Qing Gong[1*], Ying Xu[2*]

[1]The Sixth Affiliated Hospital, GMU-GIBH Joint School of Life Science, Guangzhou Medical University, No. 195 Dongfengxi Road, Guangzhou 511436, China;
[2]State Key Laboratory of Genetic Engineering, Institute of Genetics, School of Life Science, Fudan University, No. 220 Handan Road, Shanghai 200433, China;
[3]Department of General Surgery, The Sixth Affiliated Hospital, Guangzhou Medical University, Qingyuan 511500, China.

[#] These authors contributed equally to the work
* To whom Correspondence should be addressed:
Q. Gong: The Sixth Affiliated Hospital, GMU-GIBH Joint School of Life Science, Guangzhou Medical University, No. 195 Dongfengxi Road, Guangzhou 511436, China; gongqing@gzhmu.edu.cn
Y. Xu: State Key Laboratory of Genetic Engineering, Institute of Genetics, School of Life Science, Fudan University, No. 220 Handan Road, Shanghai 200433, China; yingxu2520@fudan.edu.cn; Tel: +86 21 5163 0587; Fax: +86 21 5163 0587
The authors have declared that no conflict of interest exists

This file contains:

- Supplementary Methods.
- Supplementary Figures S1-S11, and Supplementary Tables S4.

Supplementary Methods

Radiation Chimeras

Radiation Chimeras was performed as previously described (1-3). Briefly, Mice (n=6 to 10 / group, 6 weeks, female) were on the C57BL/6 background. To generate radiation chimeras, mice received a lethal dose of 9.0 Gy of ionizing radiation, followed by tail-vein injection of 10^7 bone marrow cells. To quantify reconstitution efficiency, we used congenic donors and recipients that differed at the *Ly5.1/Ly5.2* locus. Mice were maintained for 6 weeks, of which 5 weeks included antibiotics (polymyxin 13 mg/l, neomycin 25 mg/l) to allow bone marrow reconstitution without postoperative infection and inflammation.

Flow Cytometry

Reconstitution efficiency was analyzed by flow cytometric analysis of congenic Ly5.1 (CD45.2-FITC; BD Pharmingen) and Ly5.2 (Cd45.1-PE; BD Pharmingen) markers on blood cells costained for CD3e-PE-Cy5 (eBioscience) or CD11b-Tricolor (Caltag) to identify T cells and monocytes/macrophages, respectively. Whole blood was collected and stained with anti-CD45.2-FITC and anti-CD45.1-PE together with antibodies against T cell or monocytes marker (CD3-CyChrome or CD14-Tricolor). Cells were washed twice and red blood cells were lysed in ACK buffer (eBioscience). After two additional washes, cells were resuspended in FACS buffer and analyzed via FACSCalibur (BD Biosciences). Data were plotted using WinMDI2.8 software.

RNA interference and transfection

Cells at 60% confluence were transfected with 50 nM of either pre‑miR™ miRNA precursor of miR‑9‑5p (pre‑miR‑9‑5p) (AM17100, Ambion, Carlsbad, CA), or mirVana® miRNA mimic (hsa-miR-9-5p, MIMAT0000441, mmu-miR-9-5p, MIMAT0000142, Ambion, Carlsbad, CA), or miR‑9 inhibitor (miRNA Inhibitor‑9) (4464088, hsa-miR-9-5p, MIMAT0000441, mmu-miR-9-5p, MIMAT0000142 Ambion) or mirVana® miRNA inhibitor (hsa-miR-9-5p, MIMAT0000441, mmu-miR-9-5p, MIMAT0000142 Ambion) using RNAi Max (Invitrogen) according to the manufacturer's directions. In all experiments an equal concentration of a non‑targeting sequence Pre‑miR™ miRNA Precursor Negative Control #1 (pre‑miR‑NC) (AM17110, Ambion) or miRNA Inhibitor Negative Control #1 (miRNA Inhibitor NC) (4464079, Ambion) was used.

Specific shRNA targeting GSK3β was purchased from Sigma-Aldrich. Specific shRNAs targeting Akt, m-TOR, Phd1 or Phd2 were purchased from Santa Cruz Biotechnology. The IDH3α coding sequence was cloned into the CSII-CMV-MCS-IRES2-Venus vector using unique Nhe I and Age I restriction sites (4). For virus production, human embryonic kidney (HEK) 293T cells were plated in T75 flasks. At 70% confluency, cells were transfected with 20 µg of IDH3α or empty vector control construct, 10 µg of pMD2.G (envelope), and 15 µg of psPAX2 (HIV-Gag-Pol-Rev), using Lipofectamine 2000 (Thermo Fisher Scientific) according to the manufacturer's instructions. For virus concentration, the cell supernatant was spun at 114*g* for 5 min to pellet debris. The supernatant was filtered using a 45-µm low-protein binding filter (Millipore) and centrifuged at 120,000*g* for 2 hours at 4°C. The virus pellet was resuspended in DMEM, aliquoted, and stored at −80°C.

To generate IDH3α mutants Ala122Thr, Ala175Val, and Pro304His, the plasmid containing the wild-type IDH3α cDNA was used as a template in mutagenesis performed using the QuickChange® Lightning site-directed mutagenesis kit (Stratagene) according to the manufacturer's protocol.

Quantification of miRNA or mRNA expression

Total RNA from trained monocytes was extracted with TRIzol reagent according to the manufacturer's instructions (Invitrogen). One microgram of total RNA was reverse transcribed using the ReverTra Ace® qPCR

RT Kit (Toyobo, FSQ-101) according to the manufacturer's instructions. A SYBR RT-PCR kit (Toyobo, QPK-212) was used for quantitative real-time PCR analysis. The relative mRNA expression of different genes was calculated by comparison with the control gene *Gapdh* (encoding GAPDH) using the $2^{-\triangle\triangle Ct}$ method. The sequences of primers for the qPCR analysis are shown in Supplementary Table 4.

For the detection of miR-9-5p, monocytes were collected in TRIzol (Invitrogen) and were performed using the standard procedure. The miRNeasy Mini kit (QIAGEN) and TaqMan MicroRNA Reverse Transcription kit (Applied Biosystems) were used for total RNA and cDNA preparation, respectively. Power SYBR Green master mix (Applied Biosystems) was used for the q-PCR analysis on a 7900HT Fast Real-Time PCR System (Applied Biosystems). Sno-RNA U18 was used as an internal control. One-tailed *t*-tests were performed with GraphPad Prism 7.0, and a P-value ≤ 0.05 was considered significant. The sequences of primers were purchased from Tiangen Co., Ltd (Shanghai, China) and the Cat. no. are shown in Supplementary Table 4.

Microarray analysis of miRNA and mRNA

A miRCURY™ Hy3™/Hy5™ Power labeling kit (Exiqon, Denmark) was used for miRNA labeling according to the manufacturer's guidelines. Microarray analysis for miRNA profiling was conducted by Shanghai Kangcheng Technology using the miRCURYTM LNA Array system (v.18.0) according to the array manual (Exiqon, Denmark). Reverse transcription for first strand of cDNA was synthesized using an Invitrogen Superscript ds-cDNA synthesis kit (Invitrogen, USA). The ds-cDNA samples were labeled according to the manufacturer's instructions (Shanghai Kangcheng Technology, China). RNA was extracted using the RNeasy Kit (Qiagen) according to the manufacturer's protocol. RNA quality control was performed using an Agilent Bioanalyzer. RNA-seq libraries were generated using Illumina TruSeq mRNA stranded kits following Illumina protocols. Libraries were quantitated using an Agilent bioanalyzer, and the pooled libraries were sequenced with an Illumina HiSeq 4000 system using Illumina reagents and protocols. The expression levels of genes and miRNAs were compared with those of the reference RNA and were determined to be differentially expressed if the adjusted p values were <0.05 and if the fold change (FC) was >2.0.

Western blot

Proteins were lysed from cells using RIPA buffer containing 10 mM Tris–Cl, pH 8.0, 150 mM NaCl, 1% Triton X-100, 1% Na-deoxycholate, 1 mM EDTA, 0.05% SDS and fresh 1 × proteinase inhibitor. The protein concentration was determined via the Bradford method using the Bio-Rad protein assay before proteins were equally loaded and separated in polyacrylamide gels. The proteins were then transferred to a nitrocellulose filter membrane (Millipore) and were incubated overnight with indicated primary antibodies. HRP-conjugated secondary antibodies were then applied to the membrane, and the Western blot signal was detected using auto-radiographic film after incubation with Super Signal West Dura reagents (Thermo Scientific). The following primary antibodies were shown in Supplementary Table 4.

Luciferase Assay

For the 3′-UTR luciferease assay, the full-length 3′ UTR of the human IDH3α gene (1129–2655 nt, GenBank accession number NM_005530.2) was amplified using a human cDNA library as a template. To generate a mutant IDH3α 3′ UTR, site-directed mutagenesis was performed. The primers for mutagenesis were sense, 5-CTTTGTTTAAGCAGGTCCATCCTCTGTTT-3, and antisense, 5-AACAGAGGATGGACCTGCTTAAACAAAG-3. In the mutant 3′ UTR, the nucleotide sequence that is complementary to the seed region of miR-9-5p was replaced with the sequence shown in Figure 5C. The 293T cells were co-transfected with 50 ng pGL3-IDH3α 3′ UTR (wild-type or mutant) and 500 ng miR-9-5p

overexpression plasmid. The empty vector was used as a control, and the renilla plasmid pRL-TK was used as an internal control. Cells were harvested 48 hr after transfection and analyzed with the Dual-Luciferase Reporter Assay kit. Firefly luciferase values were normalized to renilla luciferase values from the same cells. The assays were repeated at least three times in independent experiments.

For the ODD luciferase assay, the ODD domain of HIF1α (GenBank accession number U59496) was fused to the 5′ end of the firefly luciferase reporter gene (5). As a control, a luciferase expression vector in which the luciferase gene is driven by the CMV promoter was used.

Chromatin-immunoprecipitation (Ch-IP) analysis

2×10^7 monocytes were isolated and trained as previously described (6). ChIP was performed on day 6, without IFN-γ priming and LPS restimulation, as previously detailed. The cells were detached from the plate with Versene and fixed in methanol-free 1% formaldehyde. Cells were then sonicated and immunoprecipitation was performed using antibodies against H3K4me3 (17-614, Millipore). After ChIP, DNA was processed further for qPCR analysis using SYBR green. Samples were analyzed by a comparative Ct method in which myoglobin was used as a negative control and H2B as a positive control according to the manufacturer's instructions. Primers are listed in Supplementary Table 4.

KDM5 Activity Assay

Monocytes were cultured as described above. Nuclear extract was performed by EpiQuik Nuclear Extraction Kit (Epigentek, OP-0002-01). Briefly, 1×10^6 cell were resuspended in 500 μl cell lysis buffer (10 mM HEPES; pH 7.5, 10 mM KCl, 0.1 mM EDTA, 1 mM DTT, 0.5% Nonidet-40, and 0.5 mM PMSF along with the protease inhibitor cocktail [Sigma]) and allowed to swell on ice for 20 min with intermittent mixing. Tubes were vortexed and then centrifuged at 12,000 $\times g$ at 4 ℃ for 10 min. The pelleted nuclei were washed twice with the cell lysis buffer and resuspended in 25 μL ice-cold nuclear extraction buffer (20 mM HEPES [pH 7.5], 400 mM NaCl, 1 mM EDTA, 1 mM DTT, and 1 mM PMSF with protease inhibitor cocktail) and incubated in ice for 30 min with intermitted sonication. Nuclear extract was collected by centrifugation at 12,000 $\times g$ for 15 min at 4 ℃. The supernatant was used immediately in a fluorometric KDM5/JARID Activity Quantification Assay Kit (Abcam), performed following the instructions of the company.

Metabolic status analysis

Culture medium was collected at day 6. The glucose and lactate concentration within the medium were determined by Glucose Uptake Colorimetric Assay Kit and Lactate Colorimetric Assay Kit respectively. NAD+ and NADH concentration were determined by NAD/NADH Quantification Colorimetric Kit from the cell lysate according to manufacturer's protocol. At least five million monocytes were lysed in 100 μL 0.5% Triton-X in PBS. Metabolite concentrations were determined by commercial assay kits for succinate, fumarate, glutamate, malate, α-ketoglutarate, following the instructions of the manufacturer. All of these assay kits were obtained from Sigma.

The oxygen consumption rate (OCR) oxygen and extracellular acidification rate (ECAR) were measured using a Seahorse XF24 technology (Seahorse Bioscience). When glycolytic metabolism was evaluated after overnight stimulation with β-glucan, monocytes (10^5 cells / well) were directly plated in 96-well Seahorse cell culture plates (200 μL final volume; Agilent Technologies). The plates were incubated at RT for 1 hour to allow even distribution of cells across the well floor. Before placing the sample plates in the Seahorse XF24, medium volume was adjusted to 175 μl in each well. Glucose at 10mM, Oligomycin at 1 μM, FCCP at 1 μM, 2-DG 100 mM, and rotenone/Antimycin A at 1 μM each, diluted in medium were injected sequentially into each well

including control wells, containing only medium, following the standard Seahorse protocol.

DNA global methylation array

DNA global methylation was measured in four biological replicates. Methylation levels were measured using the Infinium Human MethylationEPIC BeadChip Array (Illumina Inc., CA, USA), which targets more than 850,000 methylation sites. Samples were randomly plated on each chip. A 500-ng DNA sample was used to perform bisulfite conversion followed by methylation profiling according to Illumina's protocol. BeadChips were scanned with an Illumina iScan and analyzed using the Illumina GenomeStudio software. All experiments were conducted following the manufacturer's protocols. Methylation microarray data from Illumina Infinium Human MethylationEPIC BeadChip platform were analyzed using R Bioconductor package minfi (7). Raw IDAT files were loaded into R software, and raw intensity signals were preprocessed and normalized using functional normalization algorithm optimized for multicondition studies (8). The resulting normalized β values were further converted to M values [$M = \log2(\beta/(1-\beta))$] for downstream statistical analyses. Quality control was performed by manually checking quality control density plots. Probes with single-nucleotide polymorphisms were dropped as they are prone to affect methylation measurements. All probes were annotated using human reference genome GRCh37/hg19 assembly.

To identify which individual CpG loci were differentially methylated between miR9-5p overexpression and control conditions, we performed differentially methylated positions (DMP) analysis using Limma algorithm (9). The *M*-value matrix was subjected to lmfit to compute the mean difference between conditions. Differentially methylated CpG loci was defined as Benjamini-Hochberg adjusted $P < 0.05$ and absolute *M*-value difference greater than 1 ($|\Delta M\text{-value}| > 1$). To identify and visualize consistent methylation patterns within continuous genomic regions (e.g., CpG islands), we also performed differentially methylated regions (DMR) analysis using DMRcate package, which applies a Gaussian kernel smoothing to demarcate adjacent CpG sites within a genomic window (10).

Integrative analysis of expression and methylation data

To elucidate the relationship between expression level of a gene and methylation level of a CpG locus within that gene, we performed an integrative analysis combining our RNA-seq expression and methylation array data. To associate a methylation probe with a gene, we defined the putative promoter region as −2 kb to +500 base pairs (bp) of the transcription start site (TSS) and linked all CpG loci with the corresponding gene. Genes with low expression were removed. We used trimmed mean of M values (TMM) normalization across samples to adjust the difference in library size and log2 transformed the normalized expression values (11). We identified 209,063 CpG-gene pairs between 23,967 genes and 835,778 CpG sites. We then performed Pearson correlation analysis between gene expression and methylation data to identify statistically significant CpG-gene pairs. A positive correlation indicates methylation and expression change in the same direction, while a negative correlation implies changes in the opposite direction.

Deep bisulfite sequencing

Deep bisulfite sequencing (DBS) is an amplicon-based next generation sequencing technique to determine the methylation patterns of a target gene per sample. The *IDH3A* were PCR amplified from bisulfite-treated human monocytes DNA following treated with mir-9-5p inhibitor and a scrambled seq control. The *IDH3A* amplicon included 30 CpG sites. Region-specific primers with a universal adapter were used in the first-round PCR listed in Supplementary Table 4. The resulting PCR product was then amplified with sample-specific multiplex identifiers (MIDs), 454 Titanium A and B sequences, and key (TCAG) sequences. The second-round products

were purified with Agencourt AMPure XP Beads (Beckman Coulter, Krefeld, Germany) to remove remaining nucleotides and primers. DNA quality was checked on the Bioanalyzer with the DNA 7500 LabChip kit. Amplicons were quantified using the NanoDrop 2000c spectrophotometer, pooled, and diluted to 1x 10^9 molecules/µl. The bisulfite amplicon libraries (generated by emulsion PCR) were sequenced on a Roche/454 GS Junior system following the manufacturer's protocol. The sequence reads were processed using the Roche Genome Sequencer software and SFF files were further analysed using the Amplikyzer software (12). Processing of standard flowgram files (SFF) has been performed using the Amplikyzer pipeline which directly aligns the intensity sequences from SFF files to amplicon reference sequences and provides a detailed nucleotide-level analysis including the calculation of CpG methylation rates. The direct use of SFF information, without prior conversion to FASTQ format avoids information loss by rounding flow intensities. For downstream processing of Amplikyzer output files in-house R-Scripts have been used and subsequent statistical analyses of methylation rates has been performed in R (Version 3.2.2).

Quantification and Statistical Analysis

The statistical analysis was performed using Prism (GraphPad Software). 2-tailed Student's *t* test was used for comparisons between 2 groups with a normal distribution, and the differences in means among multiple groups were analyzed using 1-way ANOVA for continuous variables with normal distribution. Comparison of survival curves was carried out using the log rank (Mantel-Cox) test. For all experiments, $P < 0.05$ was considered statistically significant. Correlation analysis was performed based on Pearson's coefficient. For all data in which 2 or more independent measurements are reported, data are displayed as mean ± SEM. As indicated in figure legends, either a representative experiment or a pool is shown, and the number of repetitions of each experiment and number of experimental units (mice) is indicated.

**Supplementary Table 4 Antibody, Chemicals, Critical Commercial Assays , Animals, Primers**		
**Antibody**	SOURCE	IDENTIFIER
mTOR 1:1000	Cell Signaling Technology	#2983
Phospho-mTOR(S2448) 1:1000	Cell Signaling Technology	#5536
Phospho-S6k(T389) 1:1000	Cell Signaling Technology	#9234
Akt 1:1000	Abcam	# Ab8805
p-Akt 1:1000	Abcam	# Ab38559
GSK3β1:1000	Sigma Aldrich	#07-1413
Drosha 1:1000	Sigma Aldrich	#SAB4200151
Idh3α 1:1000	Abcam	# Ab58641
Glut1 1:1000	Abcam	#Ab115730
β-Actin 1:5000	Sigma Aldrich	#A5441
Dectin 1:1000	Abcam	#Ab140039
Tlr2 1:1000	R&D System	#MAB1530
Tlr4 1:1000	R&D System	#MAB2759
Hif 1 alpha 1:500	Abcam	#Ab16066
Hydroxy-hif1a(Pro564) 1:500	Cell signaling	#3434
Gapdh	Abcam	# Ab9485
Fh1	Abcam	#Ab233393
Histone H2B	Abcam	#Ab178462
Sdhb	Abcam	# Ab147114
Idh2	Abcam	# Ab131263
Phd1	Abcam	# Ab113077
Phd2	Abcam	# Ab13630
Pfkp	Abcam	# Ab119796
Hk2	Sigma Aldrich	#SAB2108077
Pfkm	Sigma Aldrich	#HPA002117
Pkm2	Sigma Aldrich	#SAB4200094
H3K4me3(Ch-IP)	Millipore	#17-614
CD45.2-FITC	BD pharmingen	#553772
CD45.1-PE	BD pharmingen	#561872
CD3- PerCP-Cy™5.5	BD pharmingen	# 560527
CD11b- PE-Cy™7	BD pharmingen	# 552850
ACK buffer	eBioscience	#A1049201
HRP-labled goat anti-rabbit IgG(H+L) 1:3000	Abcam	#Ab97051
HRP-labled goat anti-rabbit IgG(H+L) 1:3000	Abcam	#Ab6789
Donkey anti-Mouse IgG	Thermo Fisher Scientific	#A21202

Secondary Antibody, Alexa Fluor 488 1:3000		
Donkey anti-Mouse IgG Secondary Antibody, Alexa Fluor 555 1:3000	Thermo Fisher Scientific	#A21422
Donkey anti-Rabbit IgG Secondary Antibody, Alexa Fluor 488 1:3000	Thermo Fisher Scientific	#A21206
Donkey anti-Rabbit IgG Secondary Antibody, Alexa Fluor 555 1:2000	Thermo Fisher Scientific	#A21428
**Critical Commercial Assays**		
Nuclear and Cytoplasmic Protein Extraction Kit	Beyotime Biotechnology	#P0028
BCA Protein Assay Kit	Thermo Fisher Scientific	#23225
SYBR Premix Ex Taq	TaKaRa	RR420A
PrimeScript RTreagent Kit	TaKaRa	RR047A
ClonExpress II One Step Cloning Kit	Vazyme Biotech	#C112
Human TNF-alpha ELISA Kit	R&D System	#DTA00D
Mouse TNF-alpha ELISA Kit	R&D System	#MTA00B
Human IL-6 ELISA Kit	R&D System	#D6050
Mouse IL-6 ELISA Kit	R&D System	#M6000B
Human IL-1β ELISA Kit	R&D System	#DLB50
Mouse IL-1β ELISA Kit	R&D System	#MLB00C
KDM5/JARID Activity Quantification Assay Kit	Abcam	#Ab113464
Lactate Assay Kit	Sigma Aldrich	#MAK064
Glucose Assay Kit	Sigma Aldrich	#MAK181-1KT
Fumarate Assay Kit	Sigma Aldrich	#MAK060-1KT
Glutamate Assay Kit	Sigma Aldrich	#MAK004-1KT
Succinate Assay Kit	Sigma Aldrich	#MAK184-1KT
Magna ChIP™ G- Chromatin Immunoprecipitation Kit	Millipore	#17-611
ReverTra Ace® qPCR RT Kit	Toyobo	# FSQ-101
SYBR RT-PCR kit	Toyobo	# QPK-212
miRNeasy Mini kit	QIAGEN	#217004

TaqMan MicroRNA Reverse Transcription kit	Applied Biosystems	#4366596
Power SYBR Green master mix	Thermo Fisher Scientific	#4368577
EpiQuik Nuclear Extraction Kit	Epigentek	#OP-0002-01
Dual-Luciferase Reporter Assay kit	Promega	#E1910
Ficoll-paque	GE Healthcare	#17144003
**Chemicals**		
Rapamycin	Selleck	#S1039
AICAR	Selleck	#S1802
Metformin	Selleck	#S1950
MG132	Sigma Aldrich	# M7449
Chloroquine	Sigma Aldrich	#C6628
Ascorbate	Sigma Aldrich	#A7631
Escherichia coli LPS	Sigma Aldrich	#L2880
Oligomycin	Sigma Aldrich	#495455
FCCP	Sigma Aldrich	#SML2959
Antimycin A	Sigma Aldrich	#35410
Rotenone	Sigma Aldrich	#R8875
FCCP	Sigma Aldrich	#C2920
β-glucan	invivogen	# tlrl-bgp
GM-CSF	R&D System	# 415-ML-005
IFN-γ	R&D System	# 485-MI-100
methyl-fumarate	Sigma Aldrich	#651419
α-ketoglutarate	Sigma Aldrich	#75890
AR-A014418	Sigma Aldrich	#361549
MK2206	Beyotime	#SF2712
**Animals**	**Institute**	**Location**
miR-9-5p$^{-/-}$	Knockout mice prepared in our laboratory	Guangzhou, China
C57BL/6 mic	Cyagen Limitied	Guangzhou, China
**Mimic or inhibitor**		
mmu-miR-9-5p mimic	Ribobio	#miR10000142-1-5
mmu-miR-9-5p inhibitor	Ribobio	#miR20000142-1-5
hsa-miR-9-5p mimic	Ribobio	#miR10000441-1-5
hsa-miR-9-5p inhibitor,	Ribobio	#miR20000441-1-5
PHD1 shRNA (m)	Santa cruz Biotechnology	#sc-45617-V
PHD1 shRNA (h)	Santa cruz Biotechnology	#sc sc-45616-V
PHD2 shRNA (m)	Santa cruz Biotechnology	#sc-45538-V
PHD2 shRNA (h)	Santa cruz Biotechnology	#sc-45537-V
Akt shRNA (h)	Santa cruz Biotechnology	#sc-29195-V

mTOR shRNA (h)	Santa cruz Biotechnology	#sc-61478-V
GSK3β shRNA (h)	Sigma Aldrich	#SHCLNV-NM_002093
lenti－miR－9	SBI System Biosciences	#MMIR-9-1-PA-1
lenti-SC	SBI System Biosciences	#MMIR-000-PA-1
IDH3a shRNA (m)	Santa cruz Biotechnology	#sc-62490-SH
RNAi Max	Thermo Fisher Scientific	#13778150
QuickChange® Lightning site-directed mutagenesis kit	Stratagene	#210518
Lipofectamine 2000	Invitrogen	#11668027

**Primers for qPCR assays in Ch-IP (5' to 3')**	
**Gene**	**Epigenetic promoter primers Sequence(Sense / Antisense)**
hH2B	TGTACTTGGTGACGGCCTTA / CATTACAACAAGCGCTCGAC
hGAPDH	TACTAGCGGTTTTACGGGCG / TCG AACAGGAGGAGCAGAGAGCGA
hTNF	CGGGGAAAAGCCCTATAAAT / TCCACATTCACTGCATTCGT
hIL6	AGGGAGGACCAGAACACAGA / GAGTTTCCTCTGACTCCATCG
HK2	TTAACCACGATGGCTCACCAG / CGAAGTTGAGCCTTAGTGACC
mH2B	CTCCGGCATGGCTCAAGTAA / CCCCTTATTTGCATGCACCC
mGLUT1	CGCTACCATTTTGCTAGTGGC / CGCTACCATTTTGCTAGTGGC
mTNF	CGCGGATCATGCTTTCTGTG / GAGGACAGCAAGGGACTAGC
mGAPDH	ATCCTGTAGGCCAGGTGATG / AGGCTCAAGGGCTTTTAAGG
mIL6	CCCACCCTCCAACAAAGATTT / CAGAGAGGAACTTCATAGCGGTT
**Primers for PCR assays (5' to 3')**	
Idh3a<br>Ala-122-Thr	Forward primer :CATTTGACCTTTACACG AATGTCCGACCATGTG<br>Reverse primer :CACATGGTCGGACATTCGTGTAAAGGTCAAATG
Idh3a<br>Ala-175-Val	Forward primer :GCTCATCACCGAGGGG GTGAGCAAG CGC ATTGC<br>Reverse primer :GCAATGCGCTTGCTCACCCCCTCGGTGAGC
Idh3a<br>Pro-304-His	Forward primer :GGACATGCGAATCAC ACAGCCCTCCTGC<br>Reverse primer :GCAGGAGGGCTGTGTGATTCGCCATCTCC
mmu-miR-9-5p	Tiangen Accession No. CD202-0086
hsa-miR-9-5p	Tiangen Accession No. CD201-0142
IDH3α	Forward primer :TGGTACAAGACCCATCCCAGT<br>Reverse primer :TCAAAGATGGCAACACCGTT
IDH2	Forward primer : CACCCGCCATTACCGAGAAC<br>Reverse primer : AGCGTCTGTGCAAACCTGATA
Sdhb	Forward primer :AAGGATGAGTCCCAGGAGGG<br>Reverse primer :CAGGCGTTCCTCTGTGAAGT
Fh1	Forward primer :ATGGGGAATCACGTTGCTGTT<br>Reverse primer :GGCAATCTTTGCTGCTTTGTC
Alt1	Forward primer :CAAGGCTAAACTCACGGAGC<br>Reverse primer :GGAAATGATAGGTGCCCTCC
Alt2	Forward primer :ATGTAACAGAGCCGAAGGAGC<br>Reverse primer :ACCTGTCCAAAGCCAGAACC

Got1	Forward primer :GCTCTTTAAGGAGTGGAAAGGTA<br>Reverse primer :CCGGTGAAACTGAACATTCCAA
Got2	Forward primer :CAACCCACCTCTCAATGGGG<br>Reverse primer :CTGATGATGCGGTCAGCCAT
**Primers for DBS assays (5' to 3')**	
IDH3A-Primer1	Forward primer : AGTAGTTGTTTGGGATTGAGGATAG
	Reverse primer : TACCCCAAACTCACAAAATAAA
IDH3A-Primer2	Forward primer : TTTTTTAGTATTGTTTATATTTTTTAT
	Reverse primer :AATTCTCTCACCTAAAAAAACCTCC
IDH3A-Primer3	Forward primer : GGGGAGGAAGAAGTTTTTT
	Reverse primer :ACTCTCCTACCAACCCTC

References:

1. Janowska-Wieczorek A, et al. Platelet-derived microparticles bind to hematopoietic stem/progenitor cells and enhance their engraftment. *Blood.* 2001;98(10):3143-9.
2. Senftleben U, et al. IKKbeta is essential for protecting T cells from TNFalpha-induced apoptosis. *Immunity.* 2001;14(3):217-30.
3. Bigarella CL, et al. FOXO3 Transcription Factor Is Essential for Protecting Hematopoietic Stem and Progenitor Cells from Oxidative DNA Damage. *The Journal of biological chemistry.* 2017;292(7):3005-15.
4. May JL, et al. IDH3alpha regulates one-carbon metabolism in glioblastoma. *Sci Adv.* 2019;5(1):eaat0456.
5. Dewhirst MW, et al. Exploring the role of HIF-1 in early angiogenesis and response to radiotherapy. *Radiother Oncol.* 2007;83(3):249-55.
6. Arts RJ, et al. Glutaminolysis and Fumarate Accumulation Integrate Immunometabolic and Epigenetic Programs in Trained Immunity. *Cell Metab.* 2016;24(6):807-19.
7. Aryee MJ, et al. Minfi: a flexible and comprehensive Bioconductor package for the analysis of Infinium DNA methylation microarrays. *Bioinformatics.* 2014;30(10):1363-9.
8. Fortin JP, et al. Functional normalization of 450k methylation array data improves replication in large cancer studies. *Genome Biol.* 2014;15(12):503.
9. Ritchie ME, et al. limma powers differential expression analyses for RNA-sequencing and microarray studies. *Nucleic Acids Res.* 2015;43(7):e47.
10. Peters TJ, et al. De novo identification of differentially methylated regions in the human genome. *Epigenetics Chromatin.* 2015;8:6.
11. Robinson MD, et al.. A scaling normalization method for differential expression analysis of RNA-seq data. *Genome Biol.* 2010;11(3):R25.
12. Scala G, et al. ampliMethProfiler: a pipeline for the analysis of CpG methylation profiles of targeted deep bisulfite sequenced amplicons. *BMC Bioinformatics.* 2016;17(1):484.

Supplementary Figure S1

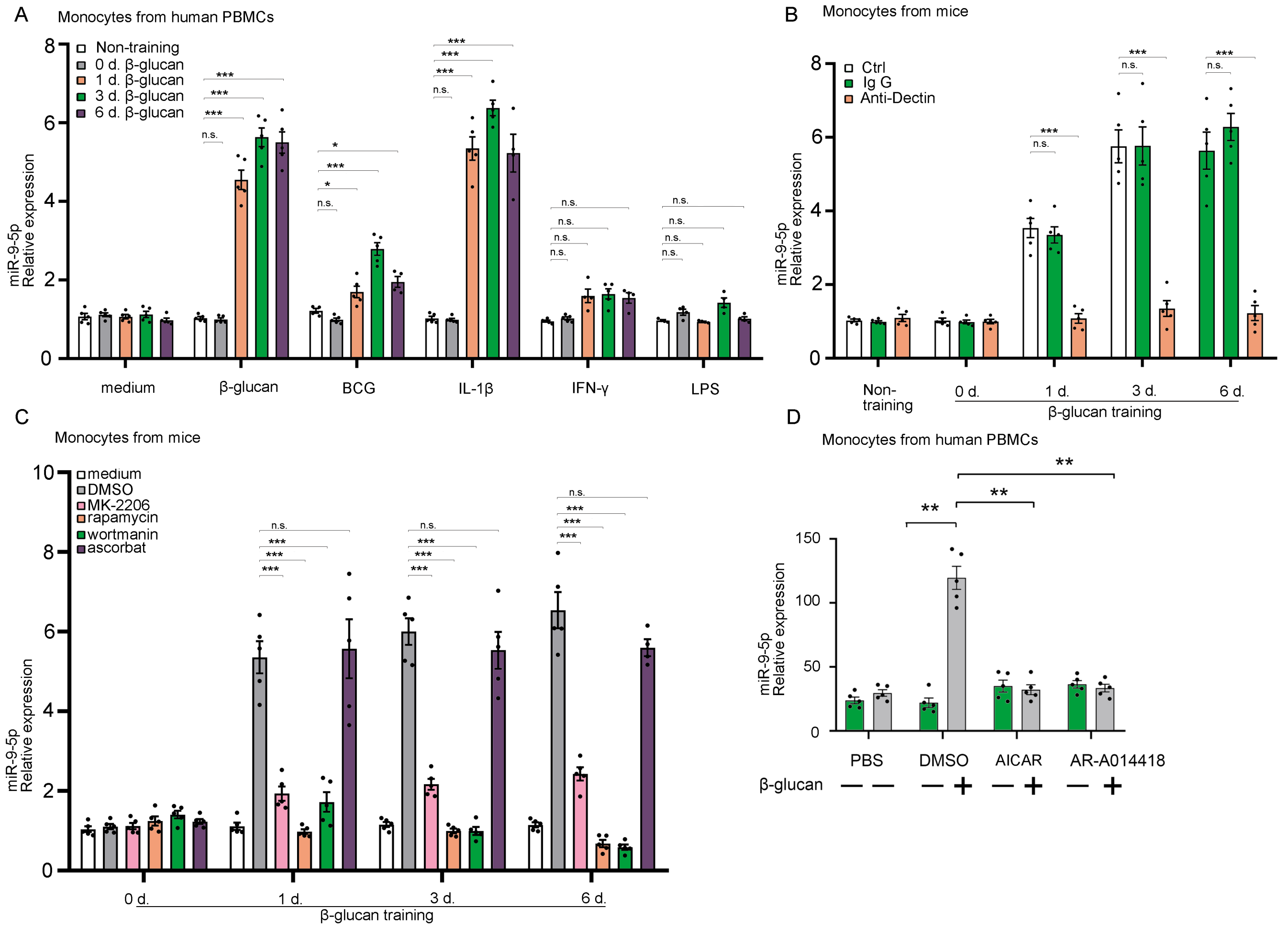

Figure S1. miR-9-5p Induction is Dependent on Dectin-1/Akt/mTOR/GSK3β Pathway in β-glucan-Induced Trained Immunity

(A) miR-9-5p levels in human monocytes stimulated with medium (as a negative control), β-glucan (5 μg/mL), BCG (BCG: monocytes = 10), IL-1β (50 ng/mL), IFN-γ (50 ng/mL), and LPS (5 μg/mL) (*n* = 3 independent experiments).

(B) Dectin-1 (20 μg/mL) blocking impaired miR-9-5p expression in β-glucan-trained monocytes from mice (*n* = 3 independent experiments).

(C) miR-9-5p expression in β-glucan-trained monocytes from mice in the presence of with DMSO (control), rapamycin (100 nM), wortmanin (30 mM), MK2206 (10 μm), ascorbate (50 μM) (*n* = 3 independent experiments).

(D) miR-9-5p level as determined by q-PCR in human monocytes trained by Medium (control), β-glucan for 24 h in the presence of DMSO (control), AR-A014418 (5 μm), or AICAR (200 nM) (*n* = 3 independent experiments).

Human PBMCs from 5 donors, mouse blood mononuclear cells from 10 WT mice, data represent means ± SEM. $^{**}p < 0.01$, $^{***}p < 0.001$ by 1-way ANOVA/Dunnett's multiple comparisons test (**A to D**). SEM, standard error of the mean. n.s. nonsense ($p > 0.05$).

Supplementary Figure S2

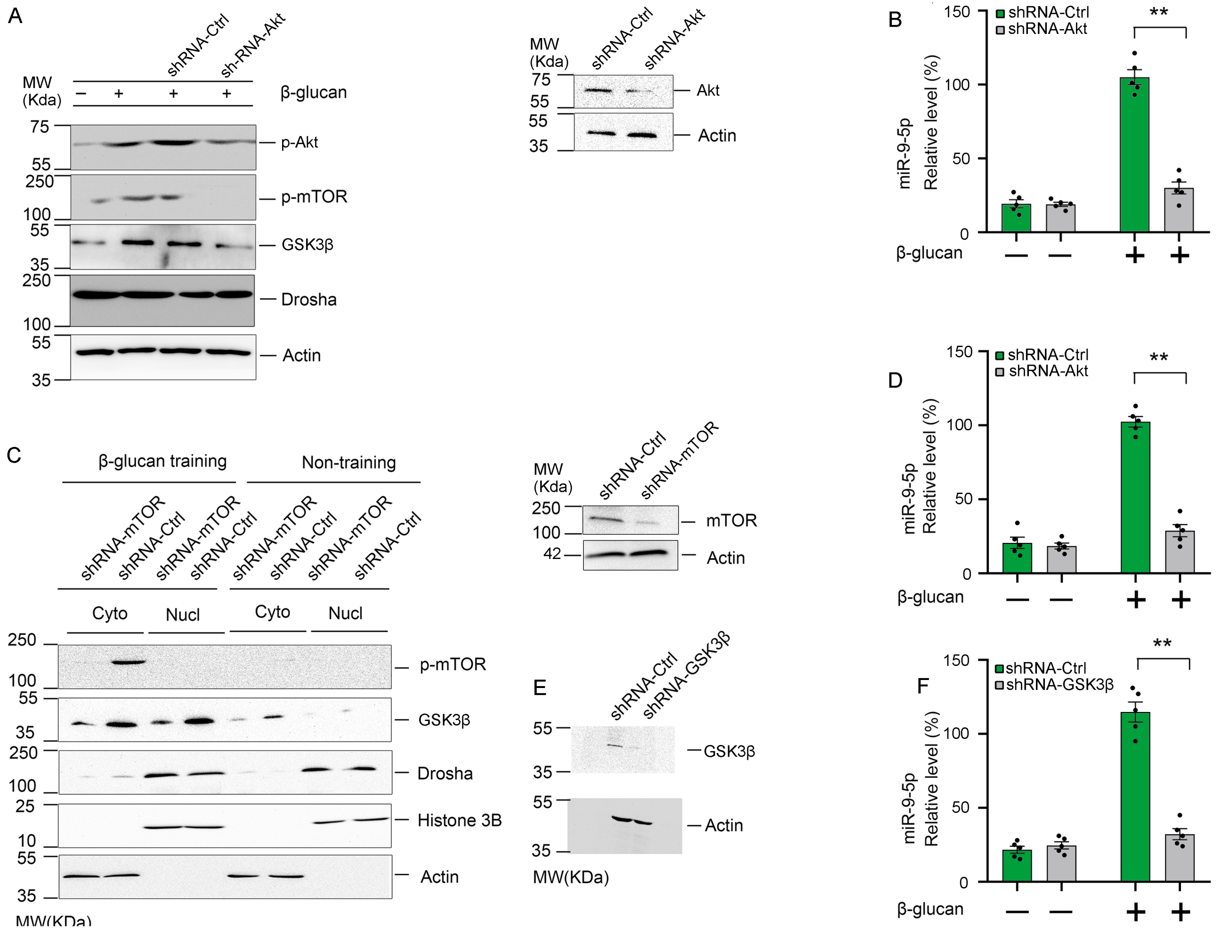

Figure S2. Genetic Inhibition of Akt/mTOR/GSK3β Pathway Impaired miR-9-5p Induction in β-glucan-Induced Trained Immunity

(A) p-Akt, p-mTOR, GSK3β, Drosha and actin protein levels as seen by immunoblot analysis of mouse monocytes treated with Medium (control), β-glucan for 24 h in the presence of shRNA for Akt or non-targeting control (*n* = 3 independent experiments).

(B) miR-9-5p level in monocytes according to **(A)** (*n* = 3 independent experiments).

(C) Total p-mTOR, GSK3β, Drosha protein, actin, Histione 3B levels as seen by western immunoblot analysis of monocytes treated with Medium (control), β-glucan for 24 h in the presence of shRNA for m-TOR or non-targeting control (*n* = 3 independent experiments).

(D) miR-9-5p level in monocytes according to **(C)** (*n* = 3 independent experiments).

(E) Immunoblot analysis of GSK3β and actin protein in monocytes treated with shRNA targeting GSK3β or non-targeting control (n=3 independent experiments).

(F) miR-9-5p level in monocytes according to **(E)** (*n* = 3 independent experiments).

Data was shown as means ± SEM, $^{**}p < 0.01$ by Two-tailed Student's t-test (**B**, **D**, **F**).

Supplementary Figure S3

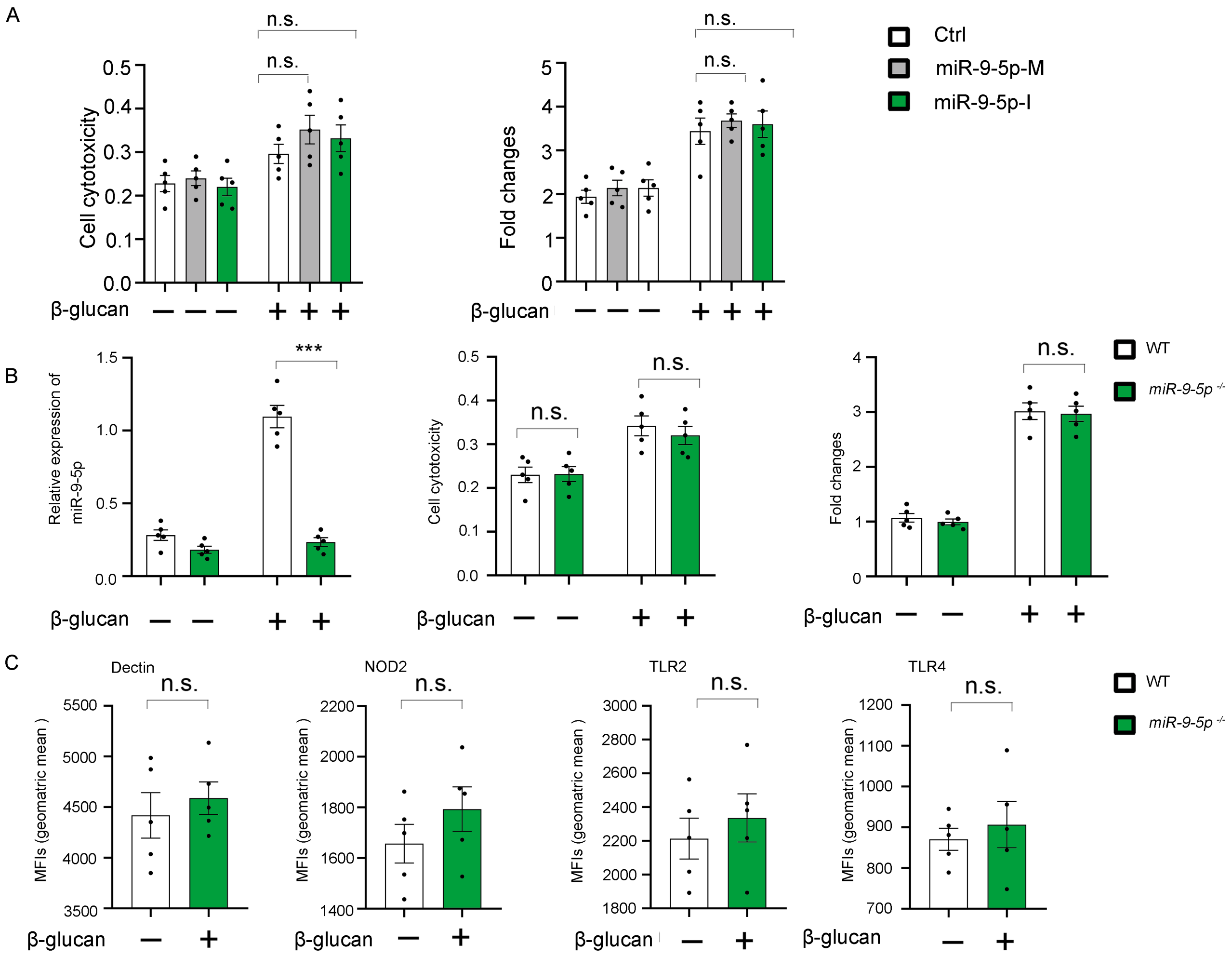

Figure S3 miR-9-5p dos not Impair Cytotoxicity, or Surface Expression of the Receptors on β-glucan-Trained Monocytes

(A) Monocytes were exposed (+) or not (-) to miR-9-5p mimics or inhibitor followed by β-glucan training according to model in **Figure 2A**, **2C**, **2E**. At day 6 and before LPS stimulation, the cytotoxicity was determined. Fold cell number was calculated by dividing live cell number in each experimental condition by the average number of Control group (*n* = 3 independent experiments).

(B) miR-9-5p expression by q-PCR, normalized to β-actin, in WT and *miR-9-5p*$^{-/-}$ monocytes under β-glucan condition. At day 6 and before LPS stimulation, the cytotoxicity was determined (*n* = 3 independent experiments).

(C) Dectin-1, TLR4, TLR2, NOD2 surface expression was analyzed by FACS in WT and *miR-9-5p*$^{-/-}$ monocytes before β-glucan stimulation (*n* = 3 independent experiments).

Data was shown as means ± SEM. Data was analyzed by 1-way ANOVA/Dunnett's multiple comparisons test (**A**); $^{**}p < 0.01$ by Two-tailed Student's t-test (**B**, **C**). n.s., not significant ($p > 0.05$).

Supplementary Figure S4

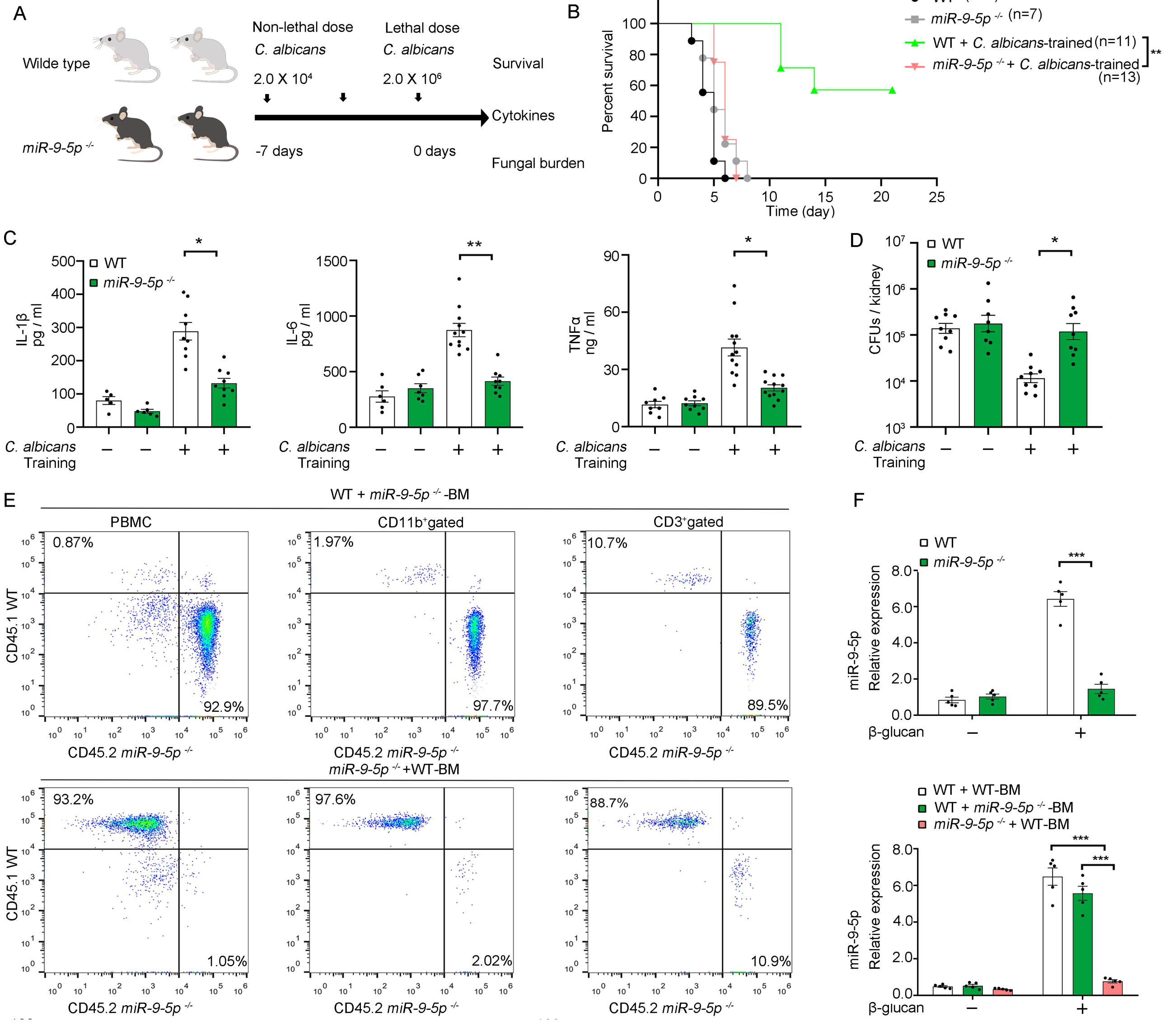

Figure S4. miR-9-5p Deletion Impairs Trained Immunity *In Vivo*.

(A) *In vivo* training mouse model via systemic infection with non-lethal dose *C. albicans* followed by a secondary lethal challenge with the same pathogen.

(B) Survival curve, according to **(A)**.

(C) Renal cytokines on day 3 post-infection (p.i.) according to **(A)**.

(D) Kidney fungal burden at indicated time points p.i. were evaluated in trained mice, following model in **(A)**.

(E) Quantification of reconstitution at 5 weeks posttransplantation by flow cytometry of total white blood cells, $CD11b^+$ cells, and $CD3^+$ cells. *miR-9-5p*$^{-/-}$ monocytes express the CD45.2 (Ly5.1) marker, whereas WT cells express the CD45.1(Ly5.2) marker according to **Fig 3 E** (n = 2 pooled experiments).

(F) q-PCR examined the miR-9-5p expression in chimeras according to **Fig 3 E** (n = 2 pooled experiments).

A pool of two experiments is shown **(B)**, including 6 to 13 mice per group as indicated, $^{**}p < 0.01$, log-rank test among radiation chimeras and WT and *miR-9-5p*$^{-/-}$ mice. Means ± SEM of two pooled experiments is shown, including at least 6 mice per condition. $^{*}p < 0.05$, $^{**}p < 0.01$ by Two-tailed Student's t-test comparing WT and *miR-9-5p*$^{-/-}$ mice (**C**, **D**); Data represent means ± SEM, $^{***}p < 0.001$ by Two-tailed Student's t-test comparing WT and *miR-9-5p*$^{-/-}$ mice (Top) or 1-way ANOVA/Tukey's multiple comparisons test (Bottom) **(F)**.

Supplementary Figure S5

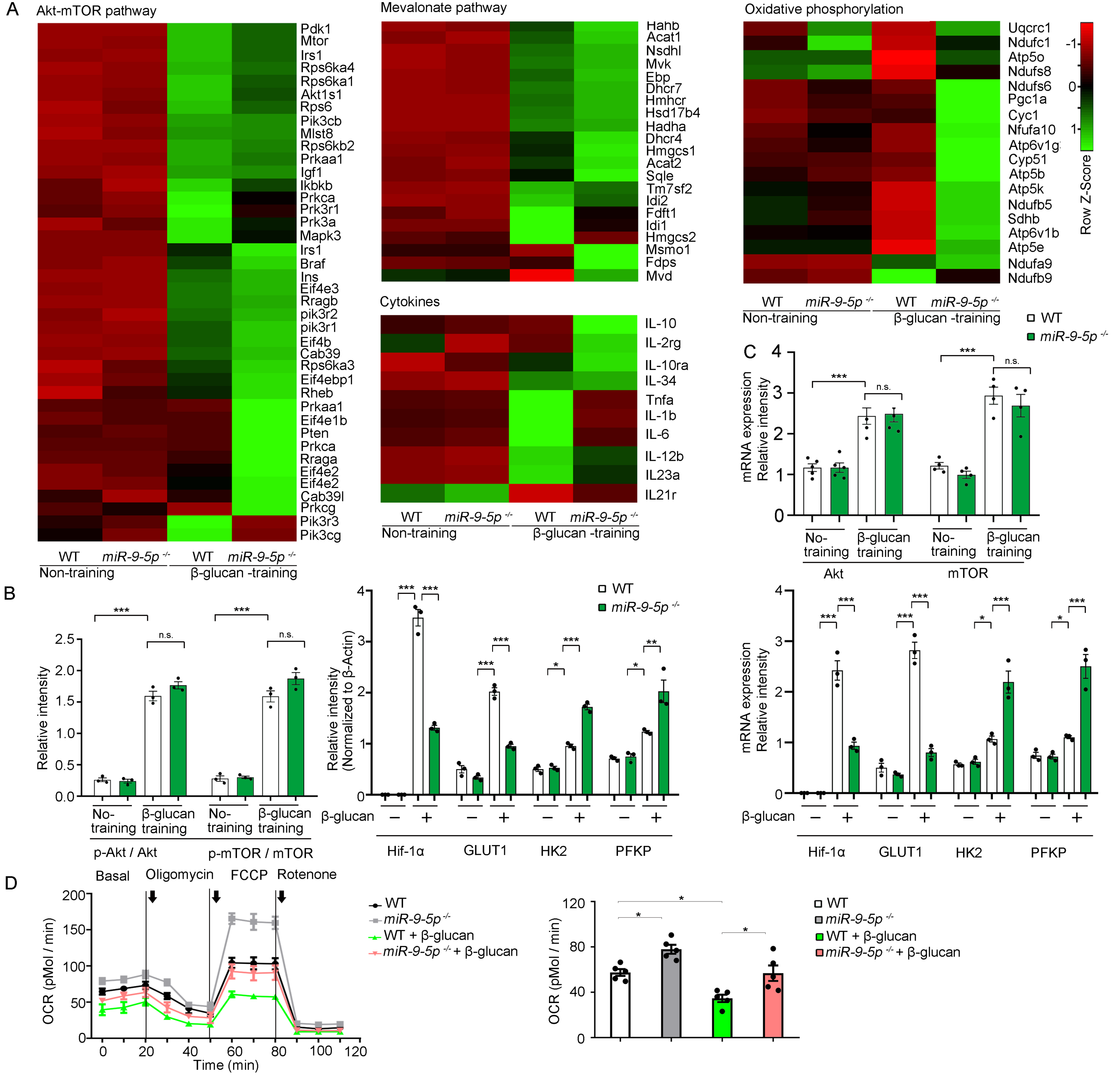

Figure S5. miR-9-5p Triggers the Switch to Glycolysis in Trained Immunity.

(A) Heatmap of genes were differently expressed in WT controls and *miR-9-5p*$^{-/-}$ monocytes, which were either unstimulated (= baseline) or stimulated for 24h with β-glucan.

(B) Quantification of western blot kinetics, related to **Figure 4D**. p-Akt / Akt, p-mTOR / mTOR, HIF-1α, HK2, GLUT1, and PFKP normalized to actin were quantified by ImageJ software. Relative band intensity is shown (*n* = 3 independent experiments).

(C) Relative amount of glycolytic genes in β-glucan-trained monocytes before LPS stimulation by q-PCR. Monocytes were exposed (+) or not (-) to β-glucan according to model in **Figure 2E** at day 6 and before LPS stimulation (*n* = 3 independent experiments).

(D) Basal oxygen consumption, oxidative phosphorylation (with oligomycin treatment), and maximum respiration capacity (with Trifluoromethoxy carbonylcyanide phenylhydrazone (FCCP) treatment) of β-glucan-trained monocytes from WT or *miR-9-5p*$^{-/-}$ mice (*n* = 4 independent experiments).

Data show the means ± SEM, $^{*}p < 0.05$, $^{**}p < 0.01$, $^{***}p < 0.001$ by 1-way ANOVA/Tukey's multiple comparisons test (**B** to **D**). n.s., not significant ($p > 0.05$).

Supplementary Figure S6

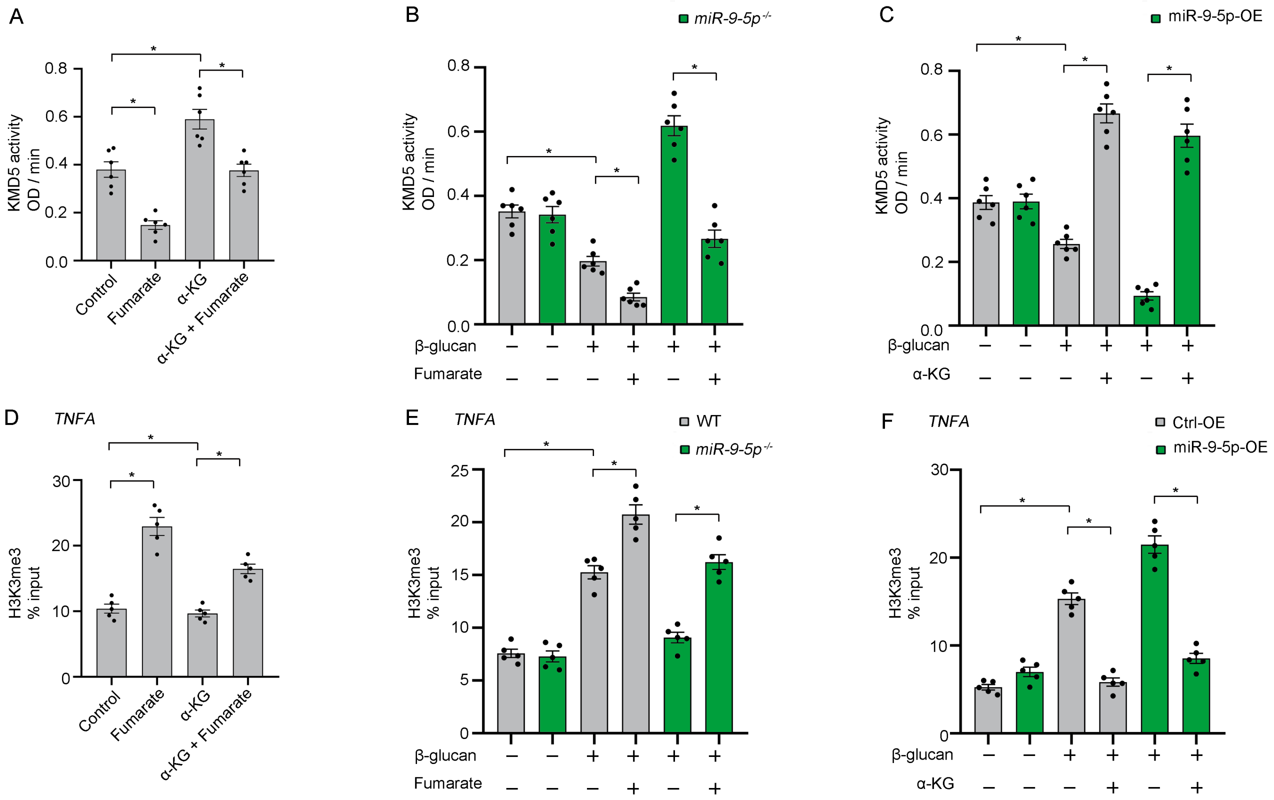

Figure S6. miR-9-5p Regulates the KDM5 Activity and Epigenetic Changes Induced via β-glucan.

(A, D) Mouse monocytes were incubated for 24 hr with fumarate (50 mM) or/and α-ketoglutarate (50 mM) after which nuclear extracts were isolated. KDM5 activity **(A)** and H3K4me3 at the promoters of *TNFA* **(D)** were determined (n = 5 independent experiments).

(B, E) The KDM5 activity **(B)** and H3K4me3 at the promoters of *TNFA* **(E)** in $miR\text{-}9\text{-}5p^{-/-}$ or WT monocytes trained by β-glucan were determined in the presence of 50 mM fumarate (n = 5 independent experiments).

(**C, F)** Human monocytes transfected with miR-9-5p-OE or Ctrl-OE were incubated for 24 hr with 50 mM α-ketoglutarate during β-glucan training after which nuclear extracts were isolated. The KDM5 activity **(C)** and H3K4me3 at the promoters of *TNFA* **(F)** was determined (n = 5 independent experiments).

The data are shown as means ± SEM, $*p < 0.05$ by 1-way ANOVA/Tukey's multiple comparisons test.

Supplementary Figure S7

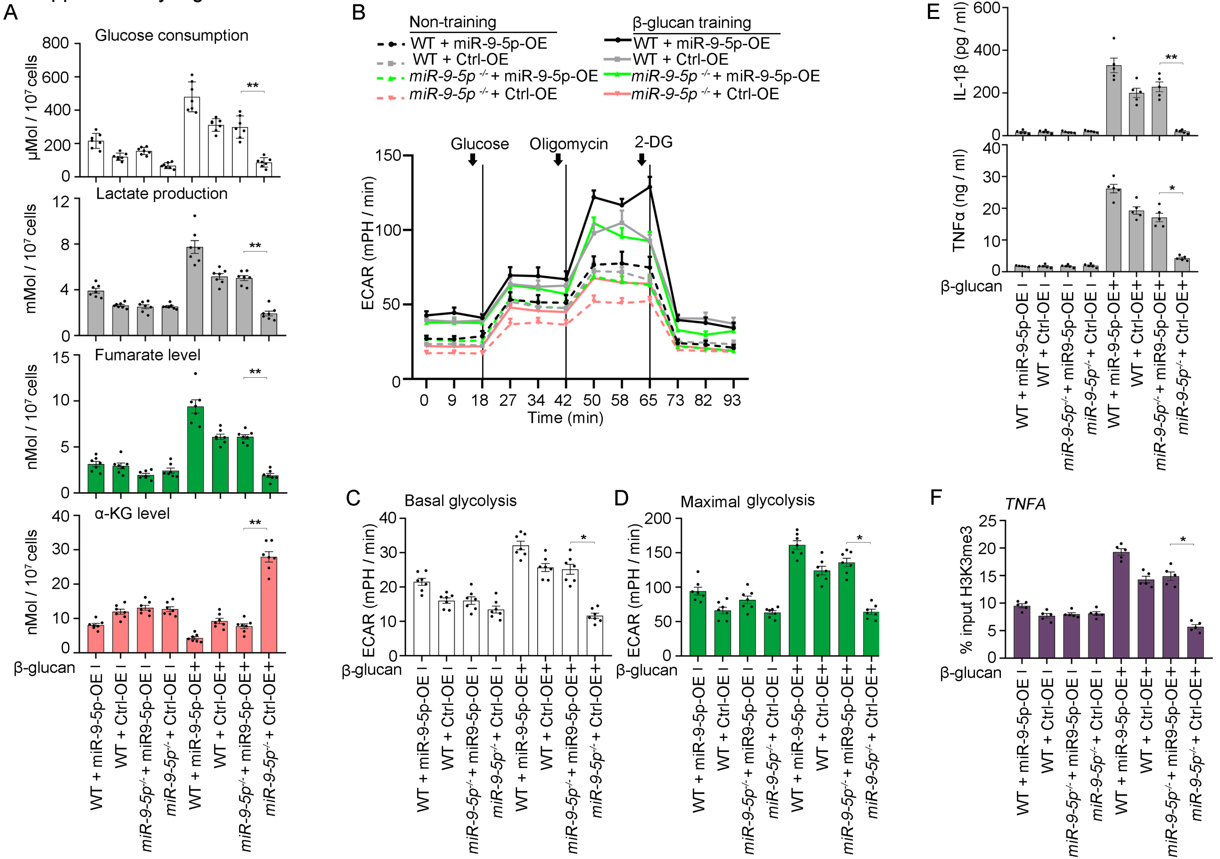

Figure S7. Compensatory Expression of miR-9-5p Rescued the Molecular, Metabolic, and Epigenetic Hallmarks of Trained Immunity Induced by β-glucan in *miR-9-5*$^{-/-}$ Monocytes.

(A) Glucose consumption levels, lactate production, and total α-KG and fumarate content were analyzed in supernatants or cell lysates according to **Figure 6G** (*n* = 5 independent experiments).

(B-D) ECAR after glycolysis stress test upon sequential addition of glucose, oligomycin, and 2-DG as indicated according to according to **Figure 6G (B)**, Basal glycolysis **(C)**, maximal glycolysis **(D)** (*n* = 5 independent experiments).

(E) IL-1β and TNFα production were analyzed in supernatants or cell lysates according to **Figure 6G** (*n* = 5 independent experiments).

(F) ChIP of H3K4me3 was performed, in which the enrichment on the *TNFA* promoter was analyzed using qPCR (*n* = 5 independent experiments) to **Figure 6G**.

In **(A, C-F)**, data represent means ± SEM. $^{*}p < 0.05$, $^{**}p < 0.01$ by 1-way ANOVA/Tukey's multiple comparisons test.

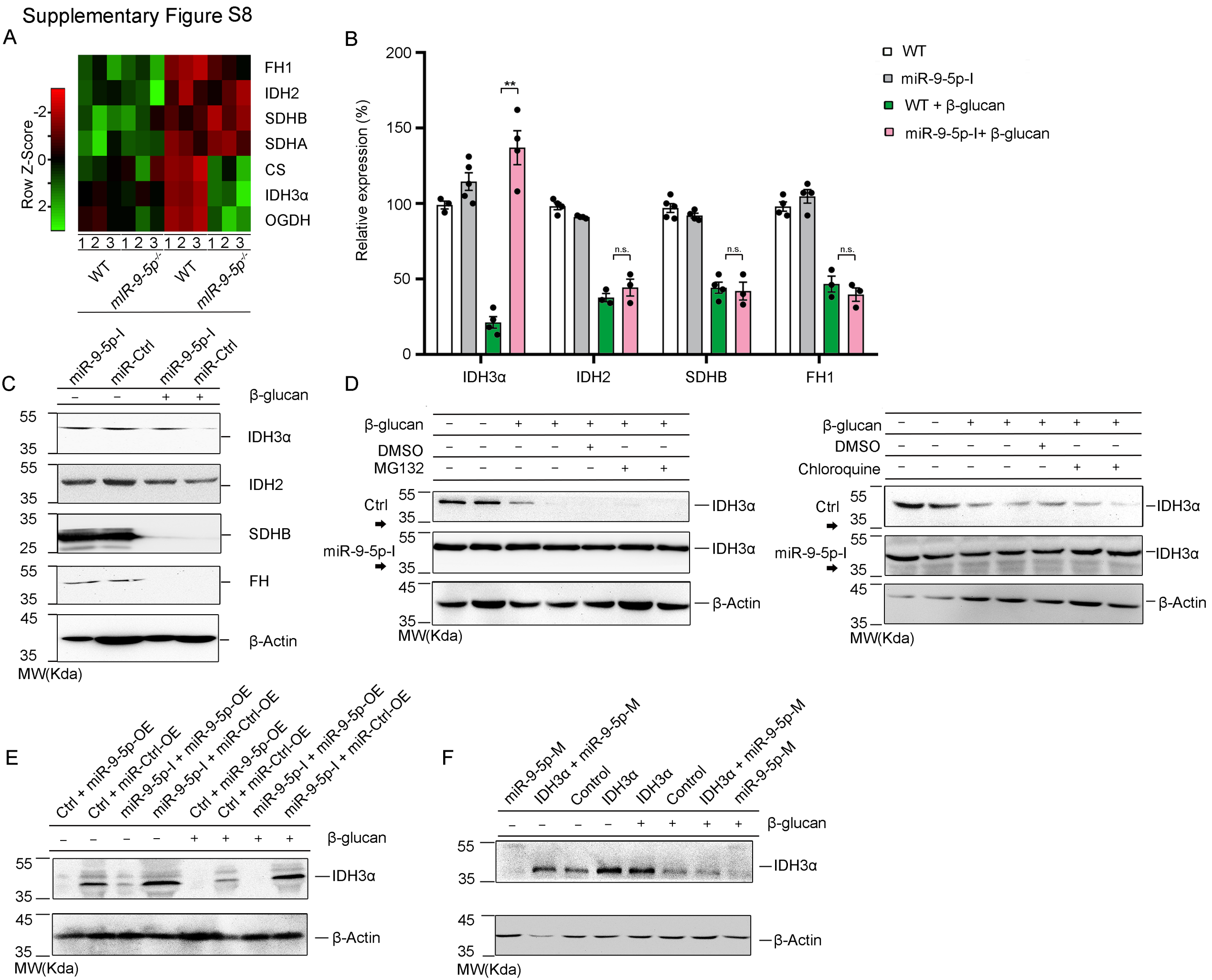
Supplementary Figure S8
A
Row Z-Score
-2
0
2
FH1
IDH2
SDHB
SDHA
CS
IDH3α
OGDH
1 2 3 1 2 3 1 2 3 1 2 3
WT
miR-9-5p-/-
WT
miR-9-5p-/-
B
Relative expression (%)
200
150
100
50
0
**
n.s.
n.s.
n.s.
IDH3α
IDH2
SDHB
FH1
WT
miR-9-5p-I
WT + β-glucan
miR-9-5p-I+ β-glucan
C
miR-9-5p-I
miR-Ctrl
miR-9-5p-I
miR-Ctrl
β-glucan
IDH3α
IDH2
SDHB
FH
β-Actin
MW(Kda)
D
β-glucan
DMSO
MG132
Ctrl
miR-9-5p-I
IDH3α
IDH3α
β-Actin
MW(Kda)
β-glucan
DMSO
Chloroquine
Ctrl
miR-9-5p-I
IDH3α
IDH3α
β-Actin
MW(Kda)
E
Ctrl + miR-9-5p-OE
Ctrl + miR-Ctrl-OE
miR-9-5p-I + miR-9-5p-OE
miR-9-5p-I + miR-Ctrl-OE
Ctrl + miR-9-5p-OE
Ctrl + miR-Ctrl-OE
miR-9-5p-I + miR-9-5p-OE
miR-9-5p-I + miR-Ctrl-OE
β-glucan
IDH3α
β-Actin
MW(Kda)
F
miR-9-5p-M
IDH3α + miR-9-5p-M
Control
IDH3α
IDH3α
Control
IDH3α + miR-9-5p-M
miR-9-5p-M
β-glucan
IDH3α
β-Actin
MW(Kda)

Figure S8. miR-9-5p Regulates the Expression of IDH3α in β-glucan-Trained Monocytes.

(A) Expression of CS, OGDH, IDH3α, FH1, SDHB, and IDH2 in β-glucan-trained monocytes from WT or miR-9-5p$^{-/-}$ mice via RNA-Seq analysis according to **Fig. 4A**.

(B) The mRNA expression of IDH3α, FH1, SDHB, and IDH2 in β-glucan-trained human monocytes (n = 3 independent experiments).

(C) Western blot analysis of IDH3α, FH, SDHB, and IDH2 expression in β-glucan-trained human monocytes (n = 3 independent experiments).

(D) Western blot analysis of IDH3α expression in β-glucan-trained monocytes from human monocytes in the presence of 10 μM MG132 (Proteasome inhibitor) or 50 μM Chloroquine (lysosome inhibitor) (n = 3 independent experiments).

(E) IDH3α expression in human monocytes with miR-9-5p-overexpression (OE), and in monocytes with or without β-glucan training in the presence of miR-9-5p-I or not (n = 3 independent experiments).

(F) Western blot analysis of IDH3α expression in β-glucan-trained monocytes overexpressing IDH3α in the presence of miR-9-5p-M or not (n = 3 independent experiments).

Means ± SEM, $^{**}p < 0.01$ by 1-way ANOVA/Tukey's multiple comparisons test. n.s., not significant ($p > 0.05$).

Supplementary Figure S9

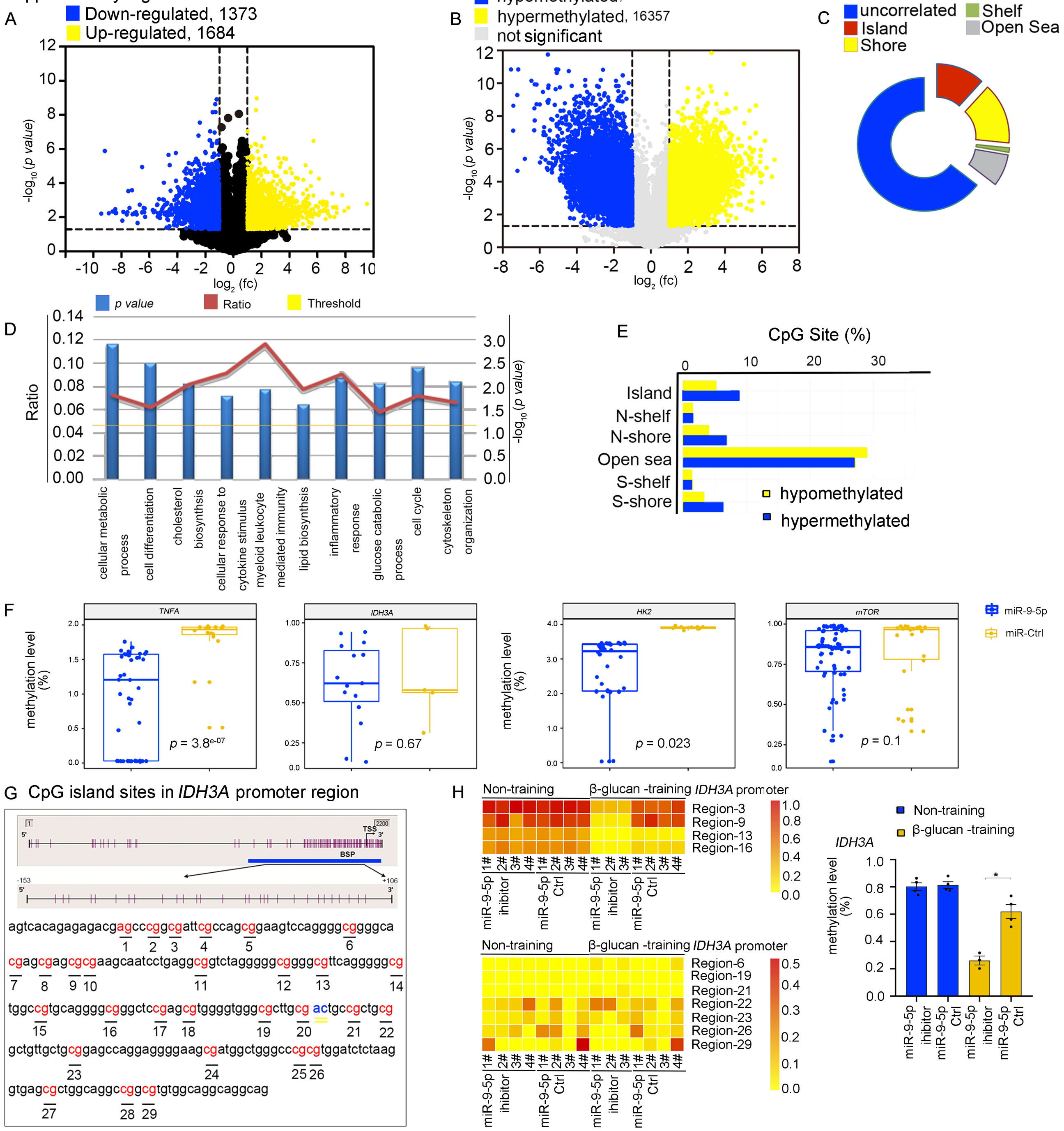

Figure S9. miR-9-5p Regulates DNA Hypomethylation

(A) Volcano plots of genes up- and down- regulated in miR-9-5p overexpression monocytes compared to the scrambled control with differential expression defined at a threshold of false discovery rate (FDR) = 0.05 and an absolute log fold change >1, *n* = 3.

(B) Volcano plots of all CpG sites differentially methylated in miR-9-5p overexpression monocytes compared to the scrambled control, *n* = 3.

(C) Graphical representation of the number of differentially expressed genes that correlated with CpG hypomethaltion, organized by CpG functional location.

(D) Pathway enrichment analysis using ingenuity pathway analysis of the genes showing correlation between mRNA expression and hypomethylation.

(E) Bar graph representation of hyper- and hypomethylated CpG sites by genomic regions in miR-9-5p overexpression monocytes compared to the scrambled control.

(F) Boxplots showing the methylation level of *HK2*, *TNFA*, *mTOR*, and *IDH3A* in miR-9-5p- or a scrambled-control- treated monocytes from 3 healthy donors.

(G) Schematic diagram of detecting DNA methylation at *IDH3A* promoter through bisulfite genomic sequencing in monocytes treated with miR-9-5p inhibitor or a scrambled seq control.

(H) *IDH3A* promoter showed partially hypomethylated in monocytes treated with treated with miR-9-5p inhibitor campared to a scrambled control. Means ± SEM, *n* = 3 to 5, *p < 0.05 by Two-tailed Student's t-test.

Supplementary Figure S10

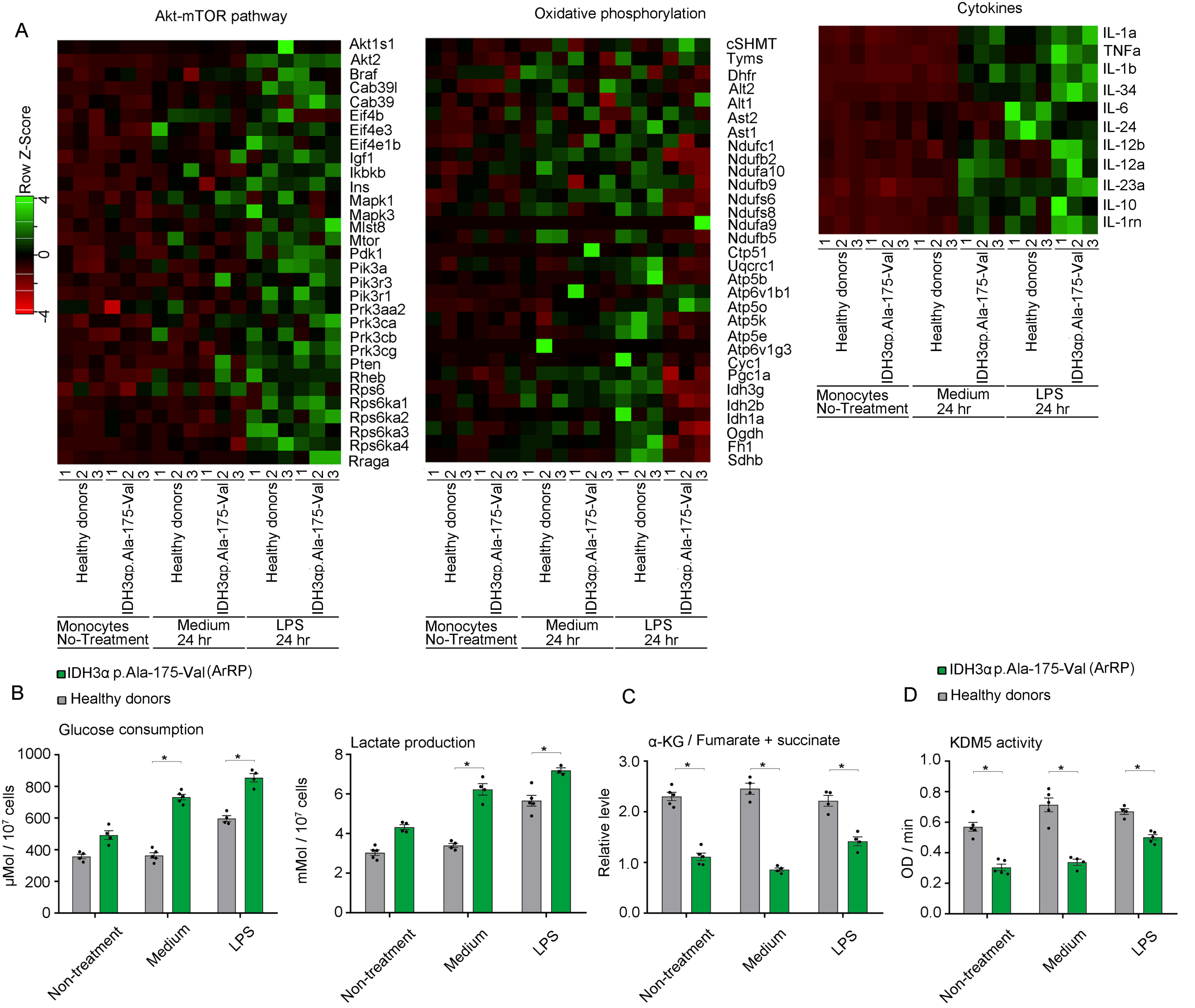

Figure S10. Heatmap of Individual Donors for Genes that Show LPS Responses in Controls and ArRP Patients with IDH3α Mutation (p.Ala-175-Val)

(A) Monocytes of 3 ArRP patient and 3 healthy volunteers were exposed to RPMI or LPS for 24 hr or left unstimulated (unstim). Expression of genes in the Akt-mTOR pathway, oxidative phosphorylation, or cytokines were determined. (n = 3 versus n = 3).

(B) Analysis of lactate production and glucose uptake in ArRP monocytes with IDH3α mutation (n = 3 independent experiments, mean ± SEM, *$p < 0.05$ by Wilcoxon signed-rank test).

(C) The relative ratio of α-KG to fumurate in ArRP monocytes with IDH3α mutant (n = 3 independent experiments, mean ± SEM, *$p < 0.05$ by Wilcoxon signed-rank test).

(D) KDM5 activity was determined in ArRP monocytes with IDH3α mutant (n = 3 independent experiments, mean ± SEM, *$p < 0.05$ by Wilcoxon signed-rank test).

Supplementary Figure S11-1

Full encropped blots for Figures

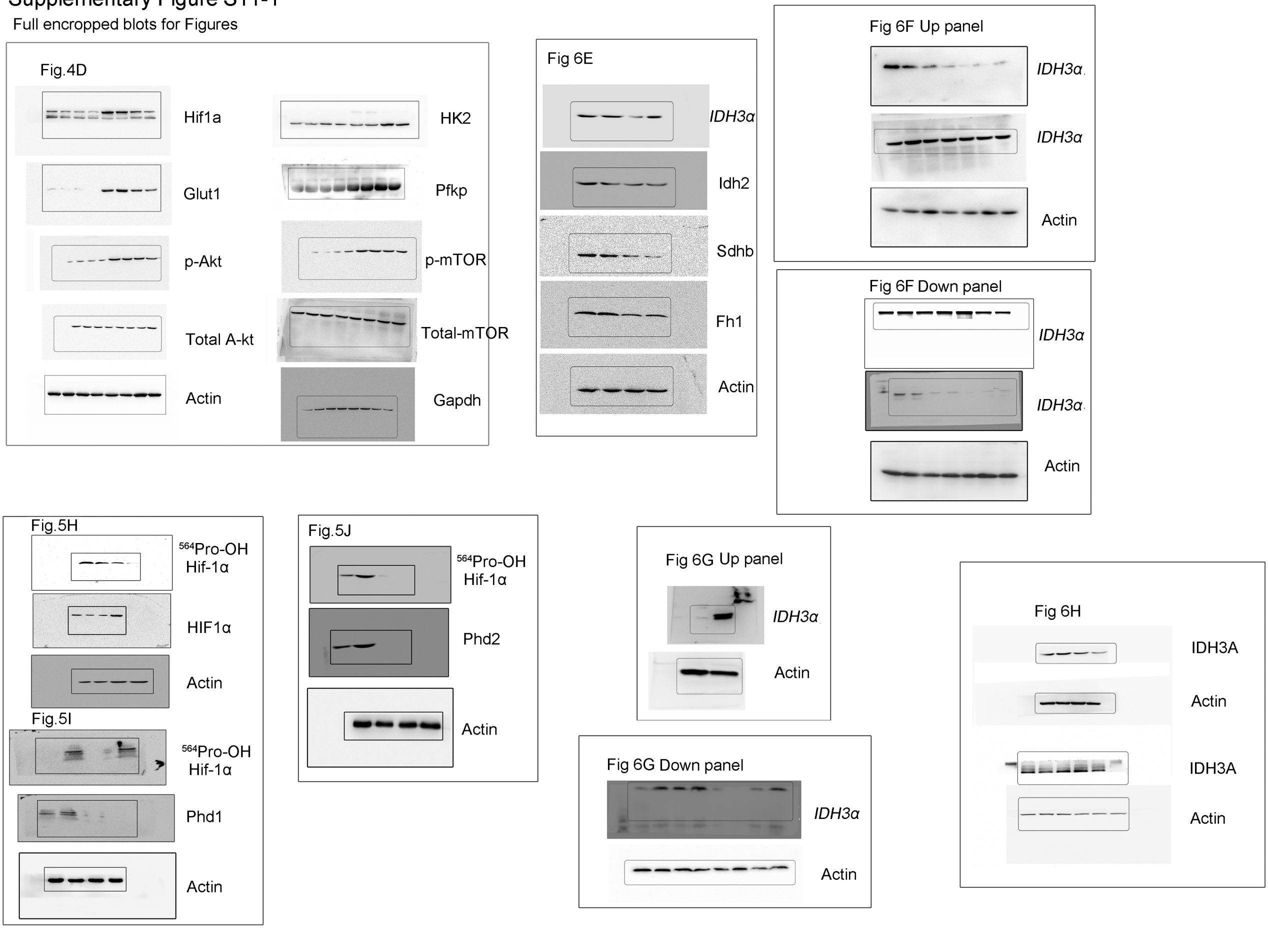

Supplementary Figure S11-2

Full encropped blots for Figures

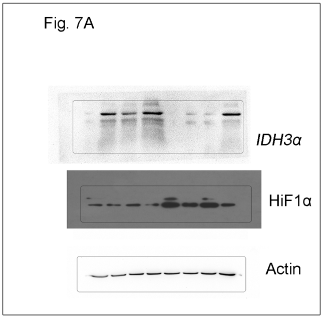

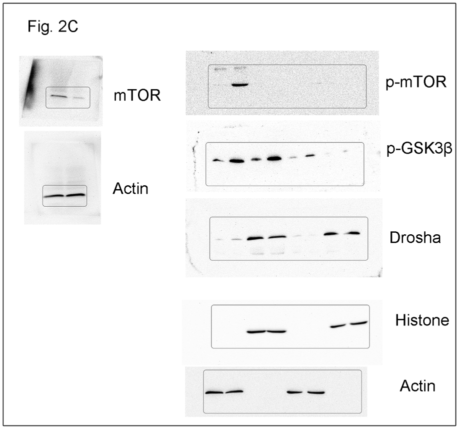

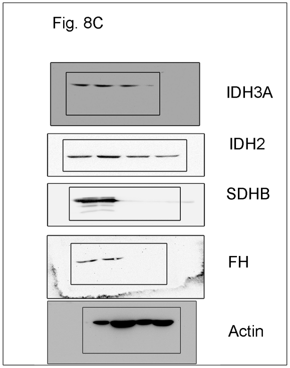

Full uncropped blots for Supplementary Figures

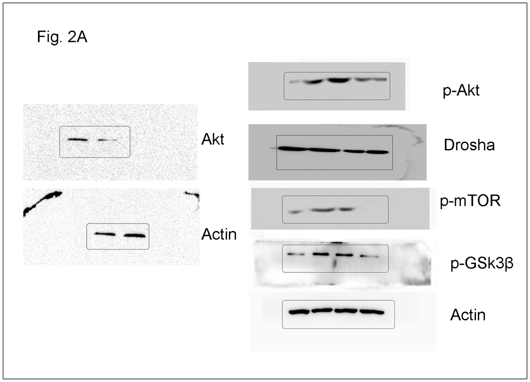

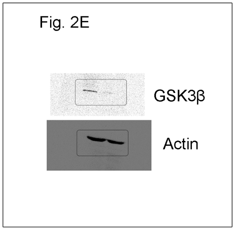

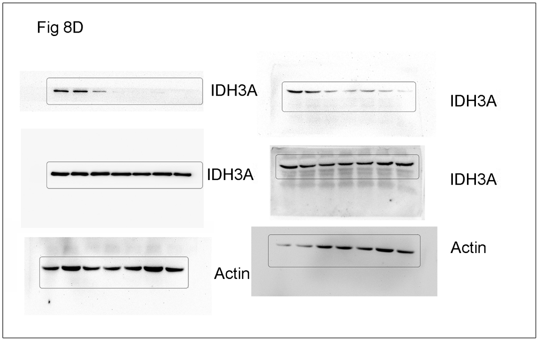

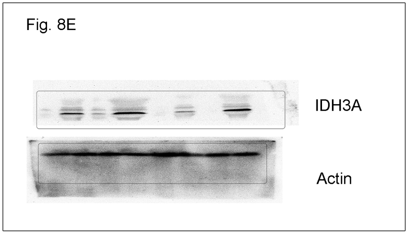

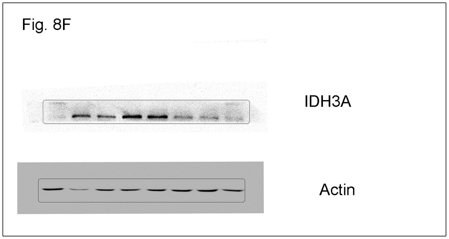

Figure S11. Full scans of uncropped images of blots.
